# From Sea to Sea: Canada's Three Oceans of Biodiversity

**DOI:** 10.1371/journal.pone.0012182

**Published:** 2010-08-31

**Authors:** Philippe Archambault, Paul V. R. Snelgrove, Jonathan A. D. Fisher, Jean-Marc Gagnon, David J. Garbary, Michel Harvey, Ellen L. Kenchington, Véronique Lesage, Mélanie Levesque, Connie Lovejoy, David L. Mackas, Christopher W. McKindsey, John R. Nelson, Pierre Pepin, Laurence Piché, Michel Poulin

**Affiliations:** 1 Institut des sciences de la mer de Rimouski, Université du Québec à Rimouski, Rimouski, Province de Quebec, Canada; 2 Ocean Sciences Centre/Biology Department, Memorial University of Newfoundland, St. John's, Newfoundland, Canada; 3 Department of Biology, Queen's University, Kingston, Ontario, Canada; 4 Research Division, Canadian Museum of Nature, Ottawa, Ontario, Canada; 5 Department of Biology, St. Francis Xavier University, Antigonish, Nova Scotia, Canada; 6 Fisheries and Oceans Canada, Maurice Lamontagne Institute, Mont Joli, Quebec, Canada; 7 Fisheries and Oceans Canada, Bedford Institute of Oceanography, Dartmouth, Nova Scotia, Canada; 8 Département de Biologie, Québec-Océan and Institut de biologie intégrative et des systémes (IBIS), Université Laval, Québec, Canada; 9 Fisheries and Oceans Canada, Institute of Ocean Sciences, Sidney, British Columbia, Canada; 10 Northwest Atlantic Fisheries Centre, St John's, Newfoundland, Canada; Dalhousie University, Canada

## Abstract

Evaluating and understanding biodiversity in marine ecosystems are both necessary and challenging for conservation. This paper compiles and summarizes current knowledge of the diversity of marine taxa in Canada's three oceans while recognizing that this compilation is incomplete and will change in the future. That Canada has the longest coastline in the world and incorporates distinctly different biogeographic provinces and ecoregions (e.g., temperate through ice-covered areas) constrains this analysis. The taxonomic groups presented here include microbes, phytoplankton, macroalgae, zooplankton, benthic infauna, fishes, and marine mammals. The minimum number of species or taxa compiled here is 15,988 for the three Canadian oceans. However, this number clearly underestimates in several ways the total number of taxa present. First, there are significant gaps in the published literature. Second, the diversity of many habitats has not been compiled for all taxonomic groups (e.g., intertidal rocky shores, deep sea), and data compilations are based on short-term, directed research programs or longer-term monitoring activities with limited spatial resolution. Third, the biodiversity of large organisms is well known, but this is not true of smaller organisms. Finally, the greatest constraint on this summary is the willingness and capacity of those who collected the data to make it available to those interested in biodiversity meta-analyses. Confirmation of identities and intercomparison of studies are also constrained by the disturbing rate of decline in the number of taxonomists and systematists specializing on marine taxa in Canada. This decline is mostly the result of retirements of current specialists and to a lack of training and employment opportunities for new ones. Considering the difficulties encountered in compiling an overview of biogeographic data and the diversity of species or taxa in Canada's three oceans, this synthesis is intended to serve as a biodiversity baseline for a new program on marine biodiversity, the Canadian Healthy Ocean Network. A major effort needs to be undertaken to establish a complete baseline of Canadian marine biodiversity of all taxonomic groups, especially if we are to understand and conserve this part of Canada's natural heritage.

## Introduction

Marine biodiversity in Canada's oceans can be assessed in several ways, each with its own attributes, limitations, and applications. First, we can report and describe past or ongoing changes in biodiversity. This descriptor establishes the relative status of marine genes, species, habitats, ecosystems, and ecological functions in Canadian waters. Second, we can describe the state of biodiversity in relation to anthropogenic activities, whether they are positive or negative [1], as judged by trends in the number of species and by potential future impacts.

Canada is at a major crossroads in its commitment to the conservation of living marine resources. On the one hand, Canada signed the Convention on Biological Diversity in Rio de Janeiro in 1992 and enacted national legislation (Oceans Act, 1996) that defines a requirement to protect marine habitat, biodiversity, and ocean health. The Oceans Act in Canada recognizes that three oceans—the Arctic, the Pacific, and the Atlantic—are the common heritage of all Canadians. Furthermore, this Act holds that conservation based on an ecosystem approach is of fundamental importance to maintaining biological diversity and productivity in the marine environment. In the Canadian context, an ecosystem approach strives to utilize a broad range of indicators (e.g. biodiversity) and measures (e.g. species richness) to develop strategies that will maintain biodiversity and function and conserve physical and chemical properties of the ecosystem [4] On the other hand, like many other regions of the world [5], Canada's oceans face numerous threats, including overfishing [2], [6], introduced species [7], habitat destruction [8], [9], alteration of food webs through removal of target species and bycatch [1], [10], [11], eutrophication and chemical loading [12], and climate change [3]. Furthermore, there is growing recognition that the diversity of life in the oceans, spanning from genes to species to ecosystems, represents an irreplaceable natural heritage crucial to human well-being and sustainable development. There is compelling evidence that Canada is on the verge of a crisis in marine biodiversity, which will only be exacerbated by complex and unanticipated effects of climate change.

Terrestrial ecologists have recognized the significance of biodiversity as an indicator of environmental health and ecosystem functioning [12], [13], [14], and the potential importance of biodiversity is now largely recognized not only by academic scientists but also by the mass media, decision makers, and the general public. However, biodiversity in marine systems has received only a fraction of the attention afforded to that in terrestrial environments [15], [16] and the link between biodiversity and ecosystem function is more tenuous [17] We know now that biodiversity in the sea—especially in the deep sea—is probably as great as on land, but far fewer marine species have been described to date [18], [19], [20]. Given current concerns about global warming, habitat degradation, and many other anthropogenic stressors, the need for protection and documentation of marine biodiversity is urgent.

To be able to assess change in the status of a nation's biodiversity, a baseline “norm” or standard is essential. The taxonomic groups targeted for the baseline reported here were determined primarily by the accessibility of datasets and the availability of authors with appropriate expertise and willingness to contribute data. Although this biodiversity assessment is not exhaustive, the inclusion of microbes, phytoplankton, macroalgae, benthic infauna, zooplankton, fish, and marine mammals encompasses many of the major groups of organisms in Canada's oceans. We must also acknowledge that some of the habitats (e.g. neritic waters, subtidal continental shelf muds) included in this baseline have already been significantly affected by human activities. The main objective of this study is to compile and identify the current number of described species of the major taxonomic groups, understanding that this list will constantly change as the biota is sampled and described more thoroughly. We hope that, over time, this list will be augmented to establish a complete inventory of known species in Canadian waters. Finally, different groups of organisms are of special interest for a variety of reasons that range from high economic value to extinction risk to exceptional species richness. We have attempted to highlight some of the key issues for these groups, although the scope of the present discussion is necessarily limited. For example, this summary discusses in some detail the marine mammal and fish species considered to be at risk of extinction, but because of the uneven information available for other groups they are rarely considered in this context, though many may also be vulnerable.

This compilation was difficult, especially given the large size and many different biogeographic provinces and ecoregions within Canadian territorial waters, which are defined here as the 12-nautical-mile contiguous coastal zone (Canada's territorial sea). Canada is bordered by the Pacific, Arctic, and Atlantic oceans, and its territorial sea covers 14.3% (2,687,667 km^2^)(Text S1) of the territorial sea area of the world. By comparison, the total territorial sea area of the 27 countries that make up the European Union (EU) is 1,008,904 km^2^, and that of the United States is only 796,441 km^2^. Further, with 16.2% of the world coastline, Canada has the longest coastline of any country. Including the mainland and offshore island coastlines, the total length of 243,791 km far exceeds the total EU countries' coastline of 143,261 km (Text S2). These numbers provide clear meaning to Canada's motto “*A Mari usque ad Mare*,” which means “From Sea to Sea.”

## Canada's three oceans: Description

Following Spalding et al. [21] on the classification of marine provinces and ecoregions of the world, Canadian oceans encompass three ocean provinces—the Arctic, the Cold Temperate Northwest Atlantic (hereafter “Eastern Canada”), and the Cold Temperate Northeast Pacific (hereafter “Western Canada”). These provinces can be further divided into 16–17 ecoregions, which represent about 7% of the 232 global ecoregions (depending on the resolution of some Arctic boundary disputes). The following section includes a description of the general circulation patterns and major physical structuring features that define the three ocean provinces.

### Arctic

The Canadian Arctic encompasses eight or nine of the 19 ecoregions in the Arctic [21]. Two Arctic ecoregions considered here, namely, the Northern Grand Banks-Southern Labrador and the Northern Labrador ecoregions, are placed in the Cold Temperate Northwest Atlantic province for purposes of this report because of the ocean circulation patterns and close linkage with the other ecoregions of this province (e.g., Gulf of St. Lawrence, Grand Banks). In general, the Canadian Arctic is covered by ice with a median normalized thickness of up to 3 m that drops to 60% of this level between mid-July and mid-October. Several independent analyses have established a declining trend in the extent of Arctic ice, amounting to −3% per decade. This trend began in the late 1970s and extends at least to the late 1990s, with a more pronounced trend in summer [22]. An animation of the change in ice extent is available online at http://nsidc.org/data/virtual_globes/images/seaice_2008_climatology_lr.mov).

**Figure 1:**
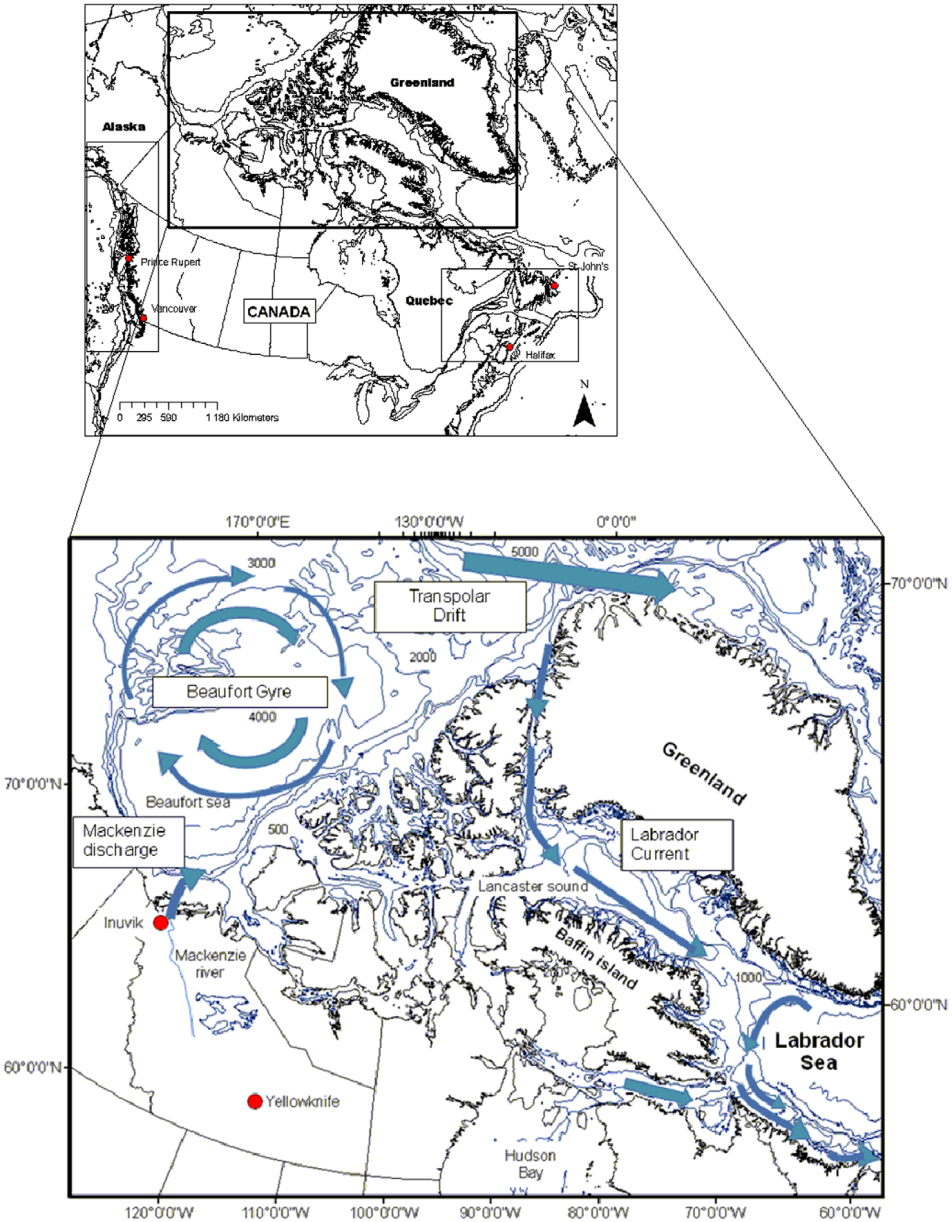
Location and general circulation patterns for the Canadian Arctic province.

The surface waters of the Canada Basin circulate in a large, clockwise rotational pattern known as the Beaufort Gyre. The circulation of the Beaufort Gyre coincides with winds of an atmospheric anticyclone centered over the Canada Basin (Figure 1) [23]. The Beaufort Sea receives about one-third of the major freshwater input in the Arctic from the Mackenzie River (340 km^3^ yr^−1^) [24]. Riverine input, especially in the Beaufort Sea, creates brackish lagoons and an estuarine habitat that supports a euryhaline community, and this input is known to affect biodiversity patterns [25] and the productivity–diversity relationship of the benthos [26]. The Canadian Arctic Archipelago forms a network of shallow channels that connect the central Arctic region with Baffin Bay. The Archipelago consists of about 16 major passages that vary from 10 to 120 km in width and from a few meters to more than 700 m in depth. However, the depth of much of the Archipelago remains uncharted. The predominant flow through the Archipelago is in a southerly and easterly direction [27]. During spring in Lancaster Sound, there is a westward current along the north side of the passage with a velocity of 22 cm s^−1^ and an eastward flow of 20 cm s^−1^ along the south side of the channel [28]. The currents through the shallow channels of the Archipelago are generally weak.

**Figure 2:**
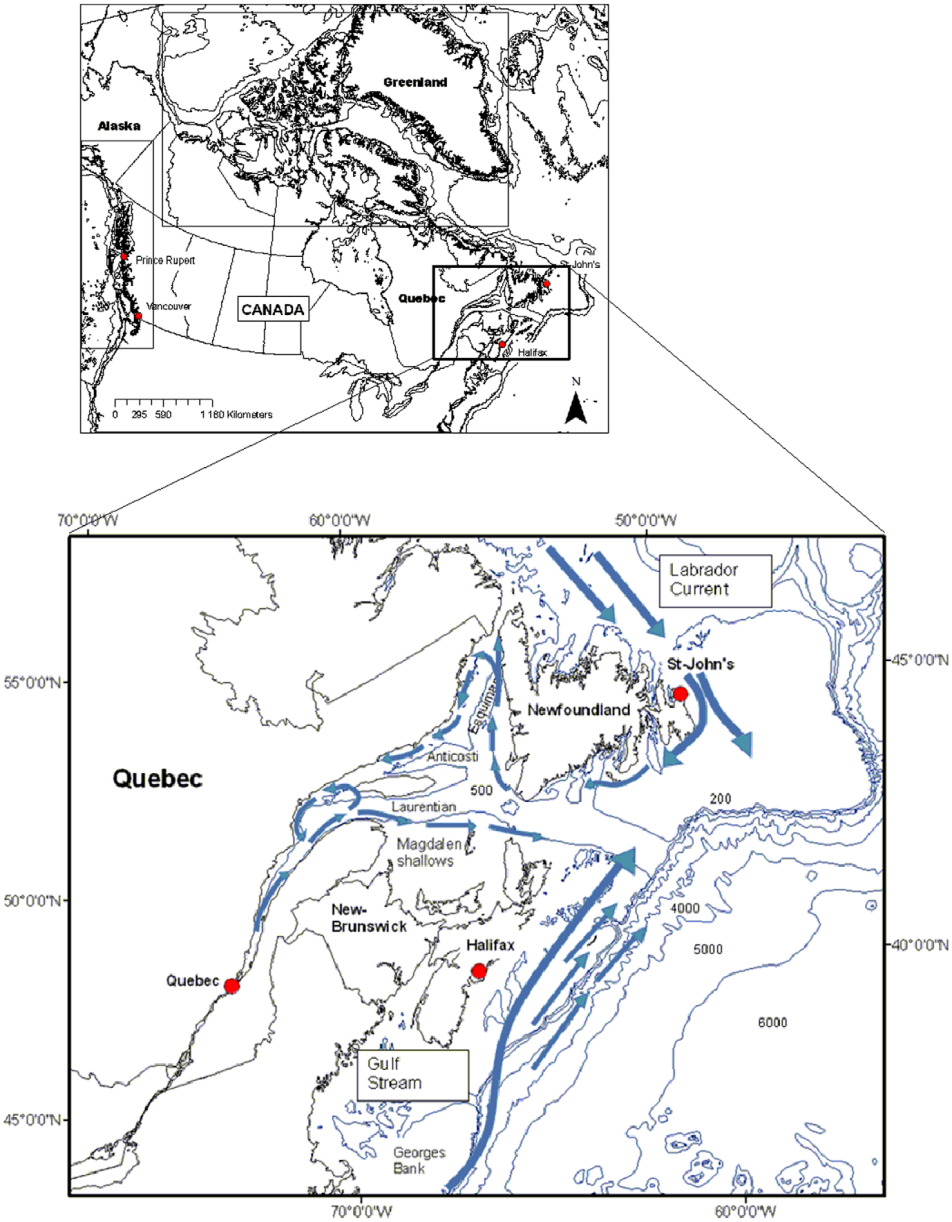
Location and general circulation patterns for Eastern Canada.

The eastern part of the Archipelago is bordered by Nares Strait, Smith Sound, Kane Basin, and Baffin Bay. Baffin Bay has a maximum depth of more than 2,300 m and is linked to the Labrador Sea (and the North Atlantic) by Davis Strait (at about 600 m depth). The Labrador Current is a continuation of the cold Baffin Island Current [29] and flows southeastwardly from Hudson Strait (the net volume of the Labrador Current is 3,170 km^3^ y^−1^) [30] and south to the Grand Banks of Newfoundland. The Labrador Current cools temperatures in the Canadian Atlantic provinces and the Gulf of St. Lawrence, and these cool waters facilitate transport of pack ice and icebergs south to Newfoundland in late winter and spring (Figure 2).

### Eastern Canada - Cold Temperate Northwest Atlantic

Eastern Canada is perhaps the best sampled area of the three Canadian provinces and includes four of the five ecoregions of this ocean province (above and beyond the two included in the Canadian Arctic, as described above) [21]. Eastern Canada is best described by partitioning it into different regions. Starting in the north, the Labrador Current flows south to the Grand Banks and enters the Gulf of St. Lawrence (GSL) through the Strait of Belle Isle and Cabot Strait [31]. To the west of Newfoundland, the GSL, a nearly enclosed shallow sea, receives about 600 km^3^ of freshwater discharges per year, roughly 70% of which come from the St. Lawrence River system [32]. The catchment area of the GSL is 6×10^6^ km^2^ with a human population density of 29.5 people km^−2^ [33]. The ice extent in the GSL peaks in March [34]. One other key defining feature is the deep (300–355 m) waters in the lower St. Lawrence Estuary that cover an area of 1,300 km^2^ and are currently hypoxic, with oxygen concentrations lower than 2.0 mg L^−1^ [35], [36].

The low-salinity water of the St. Lawrence Estuary flows northeast through the GSL to the Scotian Shelf. This region is interconnected by two sources of subpolar water, the GSL and the Labrador Current [37]. Another important feature of the area is the Gulf Stream, which enters from south of the Scotian Shelf and flows north, deflecting eastward as it flows along the Scotian Shelf and approaches Newfoundland. As it flows through these regions, it begins to broaden and sheds mesoscale warm- and cold-core water eddies.

Another important region in Eastern Canada is the Bay of Fundy. The unique funnel shape and depth of the Bay of Fundy create the highest tidal amplitude in the world at 16 m (53 ft). As an aside, a rivalry between Arctic Quebec (Ungava Bay) and the Canadian Maritimes over who has the world's highest ocean tides was declared a tie by the Canadian Hydrographic Service. The immense energy of the tides, which produce an ebb and flow that is estimated to be 2,000 times greater than the daily discharge of the GSL [38], powers a highly productive, rich, and diverse natural ecosystem that, in turn, shapes the environment, tourism, and fishing industries of the Fundy region.

### Western Canada - Cold Temperate Northeast Pacific

**Figure 3:**
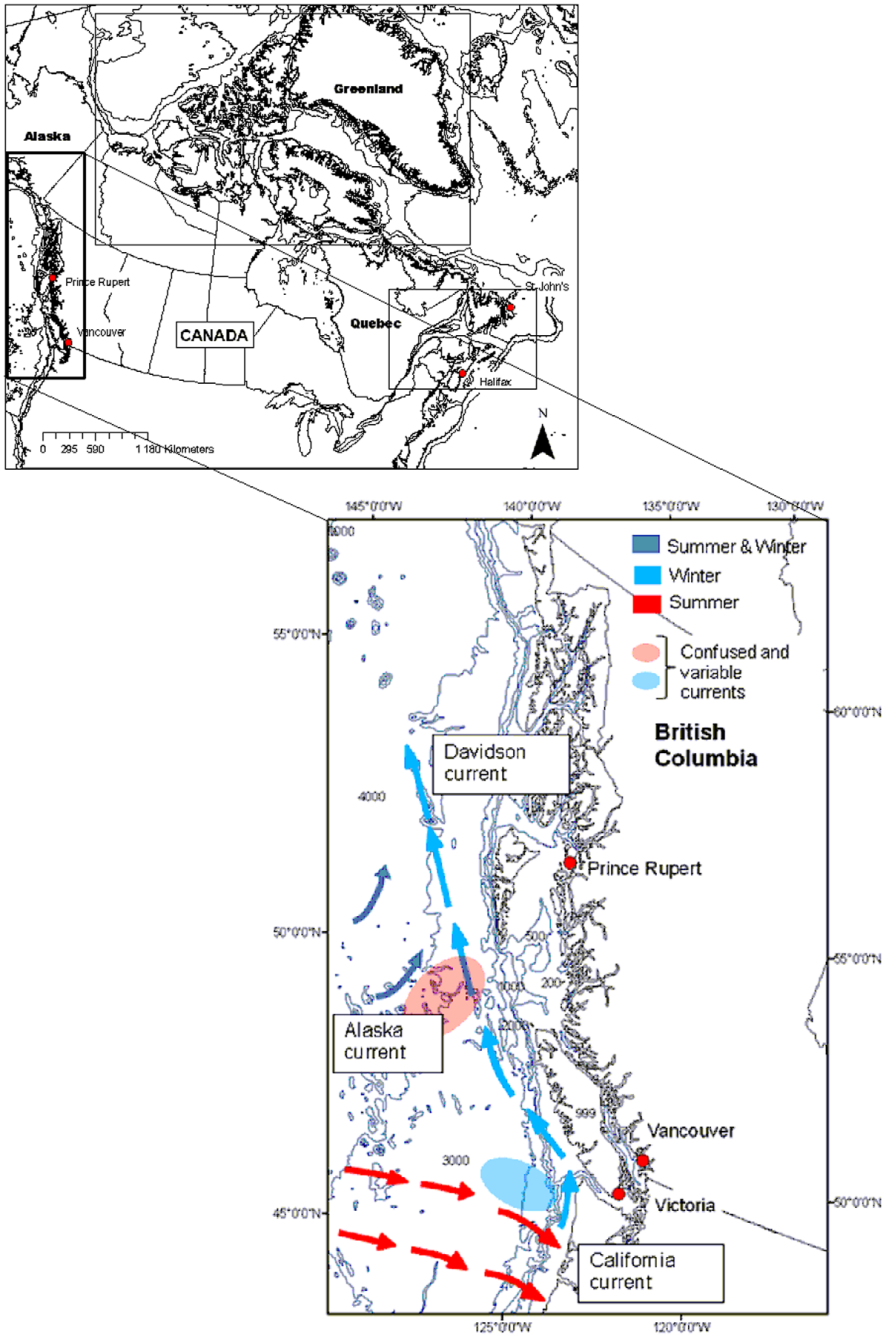
Location and general circulation patterns for Western Canada.

In Western Canada a divergence in the prevailing wind pattern causes a bifurcation in two branches of the Subarctic Current; a northern branch curves to the northeast into the Gulf of Alaska as the Alaska Current, and a southern branch curves to the southeast as the California Current. This bifurcation is variable in space, time, and intensity (Figure 3). During winter the bifurcation is abrupt and mostly confined to the southern portion (blue area in Figure 3), whereas in summer (red area in Figure 3) the current splits broadly over the region because the wind patterns are less clearly established [39]. The California Current is poorly defined and variable. In late autumn or early winter, the California Current is shifted offshore by the Davidson Current, a seasonal current that moves from 32°N northward to the coast of Vancouver Island. Thomson [39] notes that this northward flow persists to early spring (March), when the California Current moves back inshore. The circulation patterns along the coast are highly complex because the British Columbia shoreline has many inlets and fjords. Our objective here is to provide a brief description of circulation patterns and to call attention to more comprehensive views of west coast circulation [39], [40], [41], [42]. Two important facts about Western Canada are that the 4.4 million people who live in British Columbia are mostly concentrated in the cities of Vancouver and Victoria (2.6 million people combined), and that there is no ice cover along the British Columbia coastline. The latter is important because the other two marine provinces in Canada are ice covered, at least in part, either seasonally (Eastern Canada) or year-round (Arctic).

## Marine biodiversity within major taxonomic groups

The oceans are richer in phyla than terrestrial and freshwater domains. In Canada, two-thirds of the 63 major phyla are predominantly marine. About 84% of phyla occur in marine environments, compared with 72% in freshwater and 66% in terrestrial realms. The relationship is reversed at the species level, though 25% of all known species of microbiota, plants, and animals in Canada (an estimated 17,750 species) are marine [43]. Tunnicliffe [44] independently estimated that about 5,000 marine species (including algae, marine mammals, fish, and invertebrates) have been described from British Columbia waters, but this estimate did not include bacteria. Brunel et al. [45] listed 2,214 metazoan invertebrates in the Gulf of St. Lawrence. A key point to note in this context is that most metazoan taxonomists would agree that the proportion of undescribed species in the oceans is far greater than that on land, at least for phyla other than Insecta [20].

The following section presents an overview of the taxa or species observed in the three biogeographical provinces: Canadian Arctic (including the subarctic Hudson Bay System) Eastern Canada, and Western Canada. The taxonomic groups discussed are microbes, phytoplankton, macroalgae, zooplankton, benthic infauna, fishes, and marine mammals. There are several important caveats to this summary. First, there are significant data gaps, even in published information. For example, rocky intertidal environments in Canada are generally well sampled and described, but there has been no coordinated effort to integrate the many local studies that underlie this knowledge. Similarly, there has been no effort to integrate taxonomic lists for subtidal epifaunal communities in Canadian waters, but this habitat is not well sampled and the geographic coverage of such an effort would be quite limited. Second, the proportion of unknown species to validly recognized species varies with the size of the organisms. Species diversity in marine mammals and, to a lesser extent, in fishes is well known, whereas microbes are poorly known. There is also a general inverse relationship between the knowledge of diversity and both water depth and geographical remoteness. Thus, even for well-known groups such as fishes, deep-water and Arctic environments continue to yield new species. Finally, one significant constraint on this summary is the availability of data. Some datasets are considered proprietary by those who collected them, and other datasets are not available in digital format. Whether any of these resources ever enter the public domain will depend on the good will, enthusiasm, and resources of those interested in a Canadian marine biodiversity. Efforts are underway to develop an online database (The Canadian Register of Marine Species, www.marinespecies.org/carms/ but if these data are ever to become available in integrated databases, such as the Ocean Biogeographic Information System (OBIS, www.iobis.org), attitudes about data sharing will have to change, and significant resources will need to be made available to fund data rescue efforts from hard copy records in file cabinets and nonstandardized spreadsheets on computer hard drives [46]. There are scattered taxonomic lists and keys for specific pelagic ([45], [47], [48], [49], [50], [51], for a complete list see each specific taxonomic section), benthic ([45], [50], [51], [52], [53], [54], [55], [56], [57], [58], [59], [60], for a complete list see each specific taxonomic section) marine parasites [61], [62] taxa but these sources need updating and integration across regions, and often represent non-georeferenced summaries that are sometimes assembled by parataxonomists.

### Microbes - (Arctic 9,500 - with a projection up to 54,500 taxa)

Marine microbes (single-celled eukaryotes, bacteria, and archaea) form the basis of the Arctic food web. With the aid of new molecular biological techniques, it is now possible to identify the microbes that inhabit Arctic seas and estimate diversity at all taxonomic levels. Although there is no scientific consensus on what constitutes a microbial species, there is broad agreement that the various species can be separated into units of diversity that can be compared. Such operational taxonomic units are assigned at a defined level of similarity, based on the small subunit ribosomal RNA gene (SSU rRNA gene). The first surveys of Arctic microbes using these techniques were only published in 2002 [63]. Since then several studies have been carried out in the Canadian Arctic focusing on small (less than 3 microns) single-celled eukaryotic plankton (picoeukaryotes, which are poorly identified by microscopy), archaea, and bacteria [64], [65], [66], [76], [68], [69], [70]. Most recently, massively parallel tag sequencing techniques [71] have revealed that, like other oceans, the Arctic contains a remarkably diverse range of microbes [65].

By comparison, relatively little work has been carried out in waters of Eastern Canada (Atlantic) or Western Canada (Pacific). Except for the tag sequencing studies, all microbial DNA sequences are deposited in Genbank (see individual publications for accession numbers) descriptions of geographical and other data including environmental data, are being archived in the International Polar Year Polar Data Catalogue at www.polardata.ca and Microbis at http://icomm.mbl.edu/microbis/. Tag sequences are publicly available online through the visualization and analysis of microbial population structures (VAMPS) project of the Marine Biological Laboratory, Woods Hole, Massachusetts (http://vamps.mbl.edu).

The first microbial studies focused on the surprisingly abundant archaea in upper Arctic waters. An early suggestion was that these microbes originated from terrestrial soils and freshwater inflow before entering the Western Arctic via the Mackenzie River [72]. Subsequent studies found that these “nonmarine” forms were indeed abundant but that the marine populations were distinct [66], [67], [68]. The Canadian Arctic Shelf Exchange Study (CASES) project was the first to document seasonal changes in the surface and deep eukaryotic communities [73], [74]. A fundamental conclusion of these studies was that water masses are the primary structuring agent in community composition.

All studies in the Arctic to date have highlighted the importance of water masses. Much more important than depth or geography, water masses determine the makeup of microbial communities across the Arctic and presumably in other oceans [65], [75]. The implications for the effects of climate change on microbial communities are therefore enormous. As currents shift and change position relative to each other in a layered ocean, the relative position of different microbial communities to each other will change, potentially perturbing historical biogeochemical cycling patterns [65], [76], [77].

The tag sequence studies indicate that there are 300–3,000 unique bacterial “species” (at least 97% similar at the SS rRNA gene level) in separate water masses [64], with about 15 different water masses in the Arctic Ocean [78]. The total diversity of bacterial “species” in the Arctic would then be between 4,500 and 45,000 species. Clone library comparisons of bacterial diversity and eukaryotic picoplankton diversity suggest that picoeukaryotes are 10 times less diverse than bacteria [67], which means that there are probably between 450 and 4,500 picoeukaryote species in the Arctic Ocean. Similarly for archaea, which are slightly more diverse than picoeukaryotes, a good approximation would therefore be 500 to 5,000 “species.” For the Canadian Arctic this would mean a total of 9,500 to 54,500 microbe species. This estimate is similar to that of Mosquin et al. [43], but their study encompassed the three Canadian oceans. They estimated 56,568 species for this group; however, our updated Arctic evaluation suggests that this group is far more diverse.

The Arctic is changing rapidly, but our ability to predict the consequences for higher food webs and biogeochemical cycling is hampered by our poor understanding of how microbial communities interact in a complex, layered ocean. The initial goal of describing the diversity of these communities must be expanded, because there is a pressing need to identify the functional diversity within water masses and the interaction of different microbial communities. New studies are now under way in the North Atlantic as part of the Canadian Healthy Oceans Network (CHONe) of the Natural Sciences and Engineering Research Council of Canada and in the North Pacific (Lovejoy unpublished data). There is tremendous potential in such an approach [79], and closing the knowledge gap will require sustained support for acquiring relevant technological expertise, technology for high-throughput sequencing, and bioinformatics development.

### Phytoplankton (total taxa 1,657)

Marine phytoplankton are single-celled photosynthetic organisms that are adapted to live in the upper water column of oceanic and coastal regions. In a broader sense, they also encompass non-autotrophic (e.g., heterotrophic, phagotrophic, mixotrophic) microorganisms. Phytoplankton are classified following the scaling nomenclature of Sieburth et al. [80], who define pico- (smaller than 2 µm), nano- (2–20 µm), micro- (20–200 µm), meso- (200 µm–2 mm), and macroplankton (larger than 2 mm). However, most marine phytoplankton species range in size from 0.2 to 200 µm. Marine phytoplankton are responsible for less than 1% of the earth's photosynthetic standing biomass, but these microscopic organisms contribute more than 45% of the annual net primary production of the planet [81]. There are approximately 5,000 recognized phytoplankton species in the world's oceans [82], [83]; however, there may be up to 25,000 morphologically defined forms of phytoplankton [81].

Numerically, cyanobacteria, which are the only extant prokaryotic group of oxygenic photoautotrophs, represent a major portion of global marine phytoplankton. Oxygenic photosynthesis evolved only once since the Archean period, but it subsequently spread through endosymbiosis to a wide variety of eukaryotic clades [81].

The majority of phytoplankton taxa that dominate modern oceans and coastal regions are distributed among at least eight well-circumscribed major divisions or phyla [81]. However, a recent reassessment of the higher classification of eukaryotes, based on ultrastructural and molecular approaches, recognized six supergroups, which can be tentatively referred to as kingdoms [84], and the marine phytoplankton species have representatives in four of these supergroups [85].

Unfortunately, there is no exhaustive documentation of phytoplankton in Canadian marine waters, aside from two taxonomic publications from the Baie des Chaleurs [86] and the St. Lawrence system [87] for Eastern Canada. The first extensive report on phytoplankton was published [88] on waters west of Greenland, including some eastern Canadian Arctic regions. They reported a total of 89 phytoplankton species, mostly represented by large cells belonging to diatoms (48 taxa) and dinoflagellates (37 taxa). Four decades later, Hsiao [89] compiled a complete list of marine phytoplankton present in the Canadian Arctic. He recorded 354 taxa, including 244 diatoms and 86 dinoflagellates. No comparable list has been compiled for the Eastern and Western Canada ocean provinces yet. The information on the biodiversity of marine phytoplankton for Canadian waters reported here has been gathered mainly through various published and unpublished reports and scattered papers. We have exempted from this survey any old taxonomic papers that refer to descriptions of new species that have not been verified in more recent studies.

A total of 1,657 marine phytoplankton taxa have been reported from the various oceanic and coastal waters of Canada (see information sources in Table 1), with representatives in four of the six supergroups of eukaryotes [84]: Archaeplastida (chlorophytes and prasinophytes), Chromalveolata (bicosoecids, chrysophytes, cryptophytes, diatoms, dictyochophytes, dinoflagellates, prymnesiophytes, rhaphidophytes, synurids, and xanthophytes), Excavata (euglenes), and Opisthokonta (choanoflagellates). The total marine phytoplankton for Canada is dominated by stramenopiles (60%), mostly including diatoms (56%), followed by dinoflagellates (22%), and less than 5% for the other groups listed in Table 1. Surprisingly, the highest diversity of marine phytoplankton has been recorded in the coastal fringe along the Arctic Ocean. This maximum known number of phytoplankton taxa for the Arctic region includes multiple sympagic (sea-ice related) species that may have been flushed out of melting sea ice during the spring period, elevating the number of pennate diatoms (Bacillariophyceae) to a maximum of 393 taxa out of a total of 633 diatoms. The second-most-important group of Arctic marine phytoplankton includes 195 dinoflagellates, whereas other groups each represent less than 3% (Table 1). The breakdown of the Arctic into eastern, central (the Archipelago), and western Arctic reveals different levels in marine phytoplankton biodiversity (data not shown). The eastern Arctic has the greatest number of marine phytoplankton taxa at 778, followed by the western and central Arctic with 418 and 242 taxa, respectively.

**Table 1:** Numbers of extant marine phytoplankton taxa in Canada's three ocean provinces and one ecoregion (Hudson Bay).

	Pacific Ocean	Canadian Arctic	Hudson Bay	Atlantic Ocean	Canada three oceans TOTAL
**Archaeplastida/Chloroplastida**					
Chlorophyta	5	21	17	4	35
Prasinophyta	7	28	21	27	52
**Chromalveolata/Alveolata/Dinozoa/Dinoflagellata**					
Dinophyceae (dinoflagellates)	103	195	150	190	368
**Chromalveolata**					
Cryptophyceae	4	15	6	8	24
**Chromalveolata/Haptophyta**					
Prymnesiophyceae	21	26	12	41	68
**Chromalveolata/Stramenopiles**					
Coscinodiscophyceae	181	172	113	161	313
Fragilariophyceae	32	68	38	29	95
Bacillariophyceae	110	393	130	84	522
Bacillariophyta (diatoms)	323	633	281	274	930
Bicosoecida	0	5	3	3	8
Chrysophyceae	6	12	16	18	37
Dictyochophyceae	4	11	6	6	14
Rhaphidophyceae	3	2	0	1	4
Synurales	0	3	0	0	3
Xanthophyceae	0	1	0	0	1
**Excavata/Euglenozoa**					
Euglenida	2	11	8	8	20
Kinetoplastea	1	3	5	3	8
**Opisthokonta**					
Choanomonada	0	16	28	29	39
**Cyanophyceae**	0	2	2	0	4
**Incertae sedis**	3	18	31	14	42
**TOTAL PHYTOPLANKTON**	482	1002	586	626	1657

Literature used for Western Canada [189], [190], [191], [192], [193], [194], [195], Canadian Arctic [88], [89], [196], [197], [198], [199], [200], [201], [202], [203], [204], [205], [206], [207], [208], [209], [210], [211], [212], [213], [214], [215], [216], [217], [218], [219], [220], [221], [222], [223], Hudson Bay [224], [225], [226], [227], [228], [229], [230], [231], [232], [233], [234], and Eastern Canada [86], [87], [235], [236], [237], [238], [239], [240], [241], [242].

Grouped by major taxonomic ranks in the four supergroups as described by [84].

The Hudson Bay System is considered to be a subarctic region and includes Hudson Strait and Foxe Basin. It sustains a total of 586 phytoplankton taxa, mostly represented by diatoms (281 taxa) and dinoflagellates (150 taxa) and a few chlorophytes, choanoflagellates, chrysophytes, and prasinophytes.

Eastern Canada (Atlantic) is the second-most-important ocean province of Canada in terms of known phytoplankton diversity, with a total of 626 phytoplankton taxa consisting of 274 diatoms (mostly centric forms with 161 taxa), 190 dinoflagellates, 41 prymnesiophytes, 29 choanoflagellates, and 27 prasinophytes. The high diversity of small phytoplankton in the Atlantic probably reflects an increasing research effort in that region by the Maritimes and Quebec. This research focuses specifically on developing a better knowledge and understanding of these microscopic organisms rather than the better-known large diatom and dinoflagellate cells.

Finally, the Western Canada province (Pacific) offers the poorest-known diversity of phytoplankton, with only 482 taxa mostly represented by 323 diatoms, including 181 centric forms, 103 dinoflagellates, and 21 prymnesiophytes.

From this general overview, the Arctic Ocean and associated coastal fringe, which is biologically poor, contain the highest diversity of known phytoplankton, but with roughly the same proportion of centric forms as in the Atlantic and Pacific oceans. The high occurrence of pennate diatoms in Arctic marine phytoplankton is a direct consequence of melting processes of annually formed sea ice, which contributes to the release of sympagic diatoms to the upper water column. A similar situation is expected in Hudson Bay, but the research effort there has probably been far less than in the Arctic regions, thus explaining the low number of phytoplankton taxa recorded.

A last point of interest is the recent occurrence of two pennate diatoms of Pacific origin, *Membraneis challengerii* and *Neodenticula seminae*, in the Northeast Atlantic, including the Gulf of St. Lawrence [90], [91], [92]. It is important to highlight that the lack of in-depth knowledge of the biodiversity of marine phytoplankton with respect to Canada's oceanic and coastal environments reflects the immense aquatic territory of Canada.

### Macroalgae (total taxa 860 to 979)

Seaweed biodiversity encompasses benthic, mostly multicellular and macroscopic organisms assigned to the phyla Rhodophyta, Chlorophyta, and Chrysophyta, that is, the marine red, green, brown, and yellow-green algae. About 900 species in these groups are known from Canadian coastal waters. Canadian seaweed biodiversity and biogeography represent complex interactions of long-term global phylogenetic diversification (over hundreds of millions of years) and more ecologically based factors, such as climate change and biotic interactions, over shorter time scales (Pleistocene and Holocene). Modern distributions are set by this historical backdrop and the contrasting oceanographic patterns, pack ice, and climate differences among the oceans. Much of the global factual framework for understanding seaweed floras in the context of these issues was described in the seminal work of Lüning [93], major syntheses [94], and more recent reviews [95], [96]. Primary floristic synopses of the seaweed floras for the different Canadian coasts have been published for Western Canada [97], for Eastern Canada [98], [99], [100], and for the Canadian Arctic [100], .

The fundamental features and causes of Canadian seaweed species richness in Canada's three oceans are summarized as follows (see Table 2):

1. Some 650 species from Western Canada to Alaska are part of a gradually changing, species-rich flora that runs from Mexico north to the Bering Strait. Northward from British Columbia, there is increasing inclusion of species from the flora of the northwestern Pacific across the Aleutian Archipelago and a decline in species with more southerly distributions [97], [102].
2. There is a relatively species-poor Arctic flora of about 200 species [100], [101], for which the distribution extends into Eastern Canada and across the North Atlantic to northern Europe. The primary historical features that have affected this flora (including that in Greenland) are the extent to which north Pacific species have been able to colonize through the Bering Strait since the Miocene and Pleistocene glaciations and the climatic rigors of even interglacial periods [102], [103]. Of this flora, only about 20 species from the Arctic Ocean are also found in Alaska [102].
3. The flora of Eastern Canada is comparatively species poor, with about 350 described species. The primary factors that affect species richness are current climatic rigors associated with winter cold and ice, and historical constraints resulting from nonrocky shoreline south of Cape Cod, which limited southward migration of species during Pleistocene glaciations [93]. At least half the species in Eastern Canada are also distributed in western North America (Canada to Alaska), or represent species pairs that have undergone vicariant speciation [103]. Significant elements in this flora (e.g., *Chondria baileyana*) are warm temperate species with disjunct distributions south of the Bay of Fundy that became trapped in the Gulf of St. Lawrence during the postglacial hypsithermal interval.

**Table 2:** Seaweed taxa (species, subspecies, varieties) on Canada's three ocean coastlines.

Province	Chlorophyta	Phaeophyceae	Rhodophyta	Tribophyceae	Total
Canadian Arctic#	61	75	66	3	210
Eastern Canada*	90	120	130	9	350
Western Canada°	120	134	380	6	650

# loosely based on [98], [100] with inclusion of subsequent records.

*loosely based on [98], including records from the Bay of Fundy northward.

° based on [97], excluding taxa known only from Oregon but adding subsequently described taxa and some undescribed cryptic species.

The Canadian Arctic province represents distributions from the Bering Strait to Labrador; Eastern Canada extends from the Bay of Fundy to Labrador; Western Canada extends from Washington state to southeast Alaska. Values shown are conservative estimates, though totals have been rounded upward to the nearest ten.

While the numbers in Table 2 are unlikely to change substantially in the short term, new species continue to be described, based on both morphological studies and molecular methods that recognize cryptic speciation.

The coastline between northern Washington and southeast Alaska is home to a comparatively diverse flora [97]. Most species occur in strictly Canadian waters, and many also occur as far away as the Aleutian Islands and the Bering Sea [102]. Western Canada and adjacent areas are home to numerous endemic species, many of which have extremely restricted distributions (e.g., *Prasiola linearis* in the San Juan Islands and surrounding areas of Washington and Canada). Most species, however, extend beyond Canadian waters. Furthermore, absence elsewhere may be more apparent than real and simply awaits more thorough exploration and the application of relevant taxonomic expertise. Similarly, Canadian Arctic endemics are rare (*Chukchia endophytica* from two sites in Nunavut and east Greenland may qualify). This endemism may not exist at all, as cold Arctic waters and the constituent species (e.g., *Papenfussiiella callitricha*) extend well into the waters of Eastern Canada, and much of this flora extends eastward to northern Europe. *Chlorojackia pachyclados*, an apparent Eastern Canada endemic, is known from only a single site in the Gulf of St. Lawrence. Regardless, Adey et al. [104] emphasize that endemic species often have limited practical or theoretical use in characterizing large geographic regions.

Sampling intensity and taxonomic expertise severely constrain accuracy of seaweed mapping and floristic data, as well as notions of abundance and rarity. Many species are known from single descriptions or from sites with limited geographic extent. As is the case with many microscopic taxa in Canadian waters the identification of smaller epiphytes and endophytes is often problematic because of limited taxonomic expertise. Many shorelines are relatively inaccessible and have been poorly explored (e.g., Queen Charlotte Islands). The occurrence of many cosmopolitan species (especially in green algae) suggests that new species described from one region will eventually be found at more distant points when the criteria become part of more general systematic understanding, and molecular tools are more accessible than they are at present. Some species have not been resampled since their original description from a limited number or even single sites (e.g., *Chlorojackia pachyclados*). Because of the continuity of shorelines and climatic conditions with adjacent geographic areas and water circulation patterns in the Holarctic, there are few strict endemic species in Canadian waters.

The Arctic algal flora traditionally was thought to have originated from Atlantic species [105], [106]. This view was based on the high similarity of species composition between cold temperate North Atlantic and Arctic oceans. A better understanding of paleoclimates, the flora of the cold North Pacific shores, and relationships of disjunct sister taxa have resolved Dunton's [105] paradox of differing origins for shallow-water animal and algal biotas in the Arctic. Thus, while exchange has probably occurred from Atlantic to Pacific via the Arctic, this pattern is limited to a few species, and the bulk of the evidence suggests mass algal colonization in the opposite direction (e.g., [102], [103], [104]), consistent with animal biogeographic models. Furthermore, as climate change brings even limited warming of Arctic surface waters, species from the cold North Pacific are potential colonizers.

Anthropogenic introductions of seaweeds on eastern and western coasts of Canada have occurred, and seaweed species have become naturalized. On the west coast, only *Sargassum muticum* has become a prominent member of algal communities, whereas in Eastern Canada, *Fucus serratus*, *Furcellaria lumbricalis*, *Codium fragile*, and *Bonnemaisonia hamifera* have substantially changed algal communities. The prospect of increased ship traffic through the Northwest Passage, which will be facilitated by climate warming and decreased sea ice in the Canadian Arctic, will greatly increase the probability of algae invasions into the region.

### Zooplankton (total taxa 900)

Marine zooplankton are key elements of marine ecosystems, serving as the dominant conduit for the transfer of energy from phytoplankton to upper trophic levels, which in some instances can be other zooplankton. Changes in zooplankton community composition exhibit strong latitudinal and cross-shelf gradients, some of the strongest of which occur when moving from coastal areas, where extreme variations of salinity can place physiological limitations on species occurrence, to offshore areas where oceanic processes that govern distribution can dominate. Steep gradients also occur across the frontal zones associated with boundary currents, such as the California Current, the Labrador Current, and the Gulf Stream (Figures 2 and 3).

The total number of species (or higher order taxa) can be used as a rough measure of zooplankton biodiversity. However, species number alone does not include the “evenness” component of biodiversity. Generally, 80–90% or more of the total local abundance and biomass is accounted for by a much smaller number of species (1 to 20 species, depending on location and season). Also, because many zooplankton taxa have restricted depth and seasonal ranges, the total number of taxa for the three Canadian oceans in all years greatly exaggerates the diversity present at any single time and place (which is the biodiversity actually experienced by the organisms inhabiting that location). Because Canadian waters are so strongly seasonal, this problem extends well beyond zooplankton to almost every other taxonomic group.

Data collections for organisms in lower trophic levels are often acquired through short-term, directed research programs or longer-term monitoring activities with limited spatial resolution. Indices of biodiversity gathered with such restrictions can be effective in identifying changes in water masses and oceanic regimes that result from changes in environmental forcing (e.g., [107]). They may be of limited value in establishing the state of marine ecosystems or in evaluating their resilience to change in response to anthropogenic influences on food web structure because of uncertainty in the thoroughness and consistency of the information base.

Changes in spatial coverage or range (e.g., depth) of collection activity can lead to substantial changes in perceived diversity owing to differences in the water masses being sampled. Many of the changes in zooplankton diversity noted in the last several decades are attributable partly to increases in geographical coverage and depth range of collections, partly to poleward zoogeographic range extensions that have accompanied recent climate fluctuations and trends (particularly in Western Canada), and partly to recent taxonomic revisions (often including splits at the genus or species level) and the use of more complete keys in identification of routine survey samples.

#### Eastern Canada

The Canadian eastern ocean province (from Davis Strait to the Eastern Gulf of Maine, including Cabot Strait and the Bay of Fundy) has complex oceanographic influences (see Figures 1 and 2). Marine zooplankton from Eastern Canada coastal waters include members of eight phyla (Cnidaria, Ctenophora, Mollusca, Annelida, Arthropoda, Chateognatha, Echinodermata, and Chordata) with a total of 381 identified species (Table S1). The class Crustacea (phylum Arthropoda) is the most diverse mesozooplankton group, in which 88 families are represented by 269 species and members of the suborder Copepoda are responsible for about half of the group's diversity (41 families, 153 species). Cnidaria are the second-most-diverse group, in which 27 families are represented by 60 species. There have been only four species (three orders and four families) of Ctenophora identified in Atlantic waters. Most Mollusca (13 families and 16 species) are represented principally by larval stages, of which holoplanktonic Gastropoda are represented by two species of the genus *Limacina* and one species from the genus *Clione*. Many members of the phylum Annelida occur in near-surface plankton as larvae and juveniles, or as sexual epitokes or stolons, while adult stages are occasionally caught in near-bottom collections. Higher order taxa (Chaetognatha, Echinodermata, and Chordata) have low diversity; few families (two to five per phylum) are represented by a small number of species (five to eight per phylum).

Several species found in Eastern Canada were identified through intensive efforts to detail species occurrence at a few sites. Collections with similar efforts toward thorough taxonomic identification are not commonly available in other parts of the eastern region, and rare or ephemeral species are often classified into broader taxonomic categories, thereby limiting our knowledge of the overall biodiversity.

#### St. Lawrence Marine System and Hudson Bay System

The St. Lawrence Marine System (SLMS; including Gulf of St. Lawrence and the Lower St. Lawrence Estuary) and Hudson Bay System (HBS; James Bay, Hudson Bay, Hudson Strait, and the Foxe Basin) are highly dynamic estuarine systems that have distinctive physical and chemical features that influence planktonic organisms in many ways. In these environments, it is common to find a sequence of zooplankton assemblages along the salinity gradient with (i) euryhaline-freshwater species (at the riverine end), (ii) estuarine species followed by euryhaline marine species (farther downstream), and (iii) stenohaline marine species (in the marine zone) [108].

Marine zooplankton from the SLMS include 318 identified species from eight phyla (Cnidaria, Ctenophora, Mollusca, Annelida, Arthropoda, Chateognatha, Echinodermata, and Chordata), while zooplankton from the HBS include 166 species from the same phyla (Table S1). The phylum Arthropoda, which includes four different classes (Branchiopoda, Ostracoda, Maxillopoda, Malacostraca) is again the most diverse group of mesozooplankton, with 84 families represented by 245 species in the SLMS and 51 families represented by 126 species in the HBS. Members of the class Maxillopoda make up about half of the diversity of this group (41 families with 100 species in the SLMS and 29 families with 68 species in the HBS). Cnidaria are also the second-most-diverse group, with 21 families represented by 30 species in the SLMS and 16 families represented by 23 species in the HBS. There have been only five (three orders and four families) species of Ctenophora identified in the SLMS and two (two orders and two families) in the HBS. In the phylum Annelida (orders Aciculata, Canalipalpata), there are nearly two or three times more families and species in the SLMS (30 families and 96 species) than in the Atlantic (14 families and 41 species) and the Arctic (20 families and 28 species). However, only a small number of Annelida have been sampled and identified in the HBS, thereby potentially underestimating overall diversity.

#### Western Canada

Table S1 shows that Western Canada has a higher number of recorded species (481) but roughly the same number of families (127) as the other ocean provinces and regions of Canada. Nearly 40% of the Pacific mesozooplankton species are calanoid copepods (185 species in 24 families). This calanoid count is larger than that in any other Canadian region and also nearly four times larger than the number listed in Figueira's [109] earlier Canadian Pacific compilation. Some of the latter difference is attributable to post-1970 increases in the number of named species in several calanoid copepod families (Aetideidae, Clausocalanidae, Euchaetidae, Heterorhabdidae, Spinocalanidae). More are probably attributable to increased sampling intensity and to the availability and use of more complete keys in identification of routine survey samples. However, there have also been numerous northward range extensions during the last 10 to 15 years by species previously reported only from south of about 35°N. Other taxa showing elevated numbers of species in the Pacific region include siphonophores, anthomedusae, ostracods, pteropods, euphausiids, chaetognaths, hyperiid amphipods, and thaliaceans. The first three groups have all undergone extensive taxonomic revision, leading to a continuous increase in the number of identified species. However, variation in the numbers of euphausiid, chaetognath, hyperiid, and thaliacean species is clearly associated with climate-linked meridional range expansions and contractions ([110], [111], [112] Galbraith and Mackas unpublished). Pteropod species richness is higher in Western Canada than in the other ocean provinces but below that reported for regions adjoining the southern border of Western Canada (the California Current and the North Pacific Central Gyre, see Figure 3).

Taxa showing relatively low diversity in the Pacific within groups include the harpacticoid copepods (four species in three families), poecilostomatoid copepods (11 species in four families), and fully planktonic decapods (eight species in three families).

#### Canadian Arctic

This assessment of Arctic zooplankton biodiversity covers a wedge-shaped area with corners defined by Bering Strait in the west, Davis Strait in the east, and the North Pole as the apex. The southern boundary is defined by the Arctic Circle (66°N). Zooplankton diversity of the Canadian Arctic has not been exhaustively characterized. The species inventory reported here (131 families and 372 species) is very likely an underestimate, yet it is comparable to the better-studied eastern province (136 families and 381 species) ().

Overall, the relative diversity of phyla in the Arctic follows that recorded in other regions; Arthropoda are the most diverse (82 families with 292 species), followed by Cnidaria (19 families and 38 species) and Annelida (20 families and 28 species). Pacific zooplankters contribute to arthropod diversity in the western Arctic [113] but as yet do not appear to be reproductively established. Calanoid copepods dominate in the Arctic with 104 species (as this taxa does in most other areas); this is only surpassed by the calanoid diversity of the Pacific (185 species). Harpacticoid copepod diversity is notably higher in the Arctic than in any other region (65 species as compared with 25 species for the next highest); this number may be inflated because of taxonomic uncertainties. Species-level diversity of other phyla (Ctenophora, Mollusca, Chaetognatha, and Chordata) is comparable to that found in SLMS and the HBS, but lower than that found in both Eastern and Western Canada.

Seasonal ice cover, complex vertical water column structure, and inputs from both the Atlantic and the Pacific oceans create a habitat-rich environment for marine zooplankton, with continued potential for northward range expansions. As climate change modifies oceanographic conditions and as exploration of the Arctic expands, there is little doubt that the number of taxa observed in this region will increase.

### Benthic infauna (total taxa 2,127)

The seabed environment includes a great variety of physically diverse and biologically distinct habitats that collectively add to regional biodiversity. These habitats differ from each other in depth (from intertidal to the abyss), temperature, light availability, and type of substratum (ranging from hard through soft, muddy bottoms). Further, some benthic fauna lives in the sediment (infauna) or attached to the seafloor (epifauna). The benthic fauna is typically classified into size categories (macrofauna is larger than 1.0 mm, meiofauna is 0.1–1.0 mm, and microfauna is smaller than 0.1 mm). All of these organisms must be sampled with specialized gear, including trawl, box core, grab, remotely operated vehicle, and scuba diver (see Eleftheriou and McIntyre [114] for a complete list of methods) appropriate for the specific habitat and size categories. The different types of gear create a challenge in compiling species lists, because standardization is not possible and it is rarely possible to assemble a full suite of sampling gear and appropriate scientific specialists to sample the complete range of organisms at a given location. For these reasons, the compilation presented here includes only subtidal macroinfaunal species for which raw data (e.g., per grab or per quadrat) are available. This approach probably greatly underestimates the number of benthic invertebrate species in Canada's three oceans. For example, Brunel et al. [44] listed a total of 1,855 species of benthic macroinvertebrates (both epifaunal and infaunal, from all habitat types (intertidal, subtidal, soft-bottom, and hard-bottom) in the Gulf of St. Lawrence. This number represents 83.7% of all macroinvertebrate species in the Gulf of St. Lawrence and is nearly as great as the total number of infaunal species reported for all of Canada.

A compilation of published and unpublished data on the number of infaunal taxa collected with grabs and box cores in the three provinces is given in Table 3. A total of 2,127 infaunal taxa were recorded for the three oceans combined. The malacostracans and polychaetes each represent 32%, and the mollusks an additional 20%, of this total. The total number of taxa is clearly an underestimate, because many taxonomic groups are identified at a coarse taxonomic level (e.g., Nematoda). This compilation shows clear gaps in information for Western Canada and the Arctic; only about 144 samples and 243 samples were compiled, respectively for each of those provinces, far less than the 662 samples included from Eastern Canada. Additional samples (202 in total) from Lancaster Sound, Eclipse Sound, and northern and central Baffin Bay [115] and 134 samples in the Beaufort Sea area [116] were unavailable for this Canadian Arctic compilation. Amazingly, the Canadian Arctic (data are mostly from the compilation of Cusson et al. [25]) included 992 taxa, only 53 taxa fewer than were reported from Eastern Canada (1,044 taxa), where more than twice as many samples have been collected. Western Canada is also surprisingly diverse (814 taxa) considering the relatively few samples included in the compilation.

**Table 3:** Numbers of marine benthic infaunal taxa in the three ocean provinces in Canada, organized in major taxonomic groups.

	Eastern Canada	Canadian Arctic	Western Canada	Canadian three oceans
**Annelida**	343	313	347	693
Polychaeta	342	306	331	673
**Arthropoda**	323	430	242	752
Malacostraca	291	385	203	673
Maxillopoda	16	3	25	34
Ostracoda	3	31	9	40
**Brachiopoda**	3	4	1	5
**Chordata**	14	21	0	32
**Cnidaria**	36	9	5	44
Anthozoa	17	7	4	24
Hydrozoa	18	2	0	19
**Echinodermata**	52	35	24	87
Asteroidea	14	11	2	22
Holothuroidea	14	7	7	22
Ophiuroidea	17	14	13	33
**Echiura**	1	1	1	3
**Ectoprocta**	8	3	0	10
**Hemichordata**	2	0	1	2
**Mollusca**	223	154	173	432
Bivalvia	92	70	92	185
Gastropoda	116	73	116	215
**Nematoda**	1	1	1	1
**Nemertea**	5	3	6	10
**Platyhelminthes**	3	1	2	4
**Porifera**	6	4	0	13
**Sipuncula**	8	10	8	20
**Others**	16	3	3	19
**Total**	1044	992	814	2127

Literature used for the compilation: [9], [25], [243], [244], [245], [246], [247], [248], [249], [250], [251], [252], [253], [254], [255], [256], [257], [258], [259], [260], [261], [262], [263], [264], [265], [266], [267], [268].

**Figure 4:**
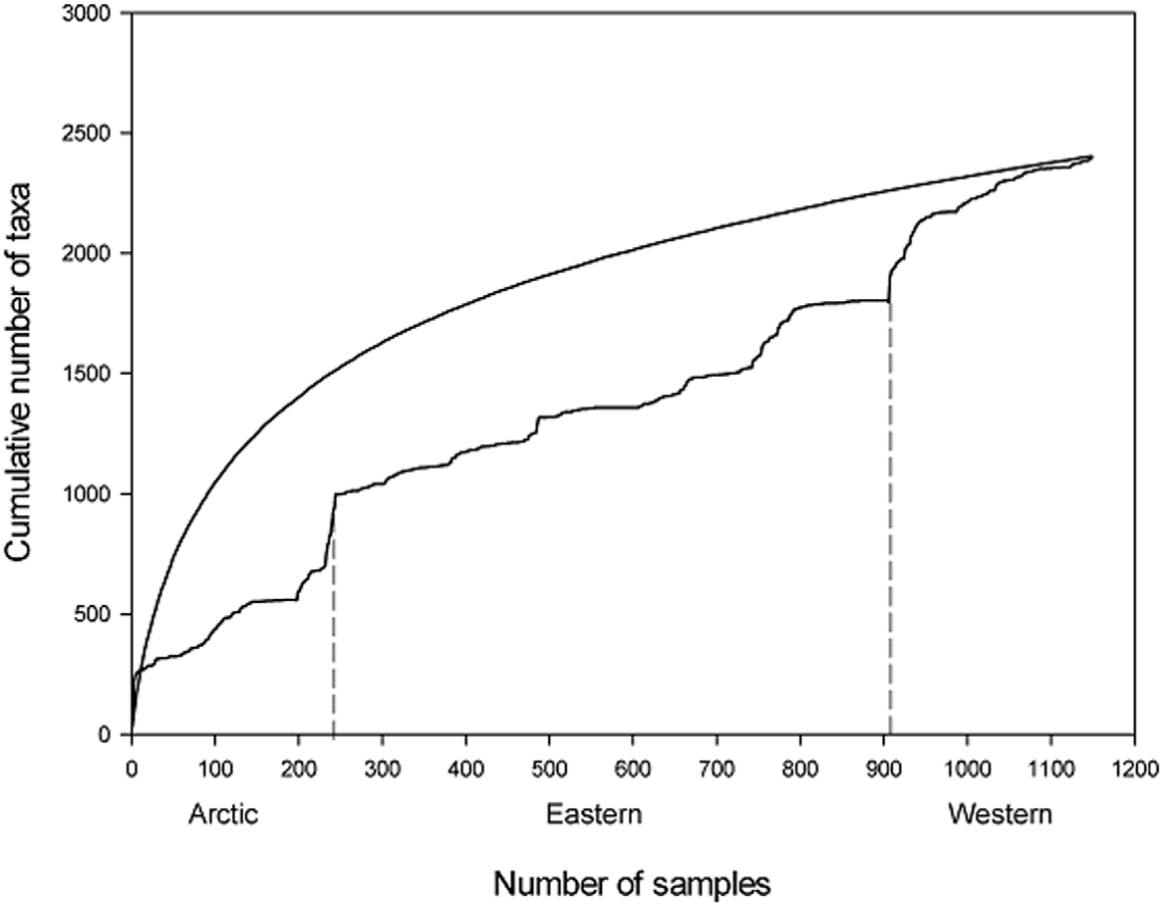
Plot of taxa accumulation curves of infauna for the three Canadian ocean provinces. The top curve represents the rarefaction curve for the combined three provinces and the lower curve represents samples accumulated in stations within each ocean province (Canadian Arctic, Eastern Canada, and Western Canada).

Figure 4 represents the taxa accumulation curves for infauna from the three ocean provinces. The continuing rise of the global taxa accumulation curve suggests that the infaunal community contains many more species. The taxa accumulation curves for each province suggest that the Arctic province and Western Canada are undersampled. Note the rapid increases in number of taxa as samples are taken from Eastern to Western Canada. Furthermore, the abrupt increases in the number of taxa in the Arctic clearly highlight that this area of Canada contains many more species that have not yet been discovered. The number of samples compiled represents only 248 m^2^ of seafloor in the three Canadian provinces. Eastern Canada has the best coverage with 178 m^2^ of seafloor sampled, while Western Canada has very little coverage (20 m^2^) and the Arctic has 53 m^2^.

In the Canadian Arctic, Cusson et al. [25] compiled data from 219 stations to generate a total list of 947 species or taxa, which represented 229 families, 68 orders, 29 classes, and 15 phyla. Arthropoda and Annelida represented 43% and 32%, respectively, of all Arctic macrofaunal species. Benthic composition varied from west to east across the study region, with an average composition of 37% Annelida and 31% Arthropoda. In their study, Cusson et al. [25] found the lowest taxa richness in the Hudson Bay ecoregion (followed by James Bay and the Beaufort-Mackenzie areas) and the highest values in the highly dynamic ecoregions of Ungava Bay and Davis Strait. The low primary production observed in Hudson Bay [117] could explain the small number of taxa. Salinity explained a large portion of the variance in number of taxa in the Beaufort-Mackenzie and James Bay ecoregions [25], [26].

The major threat for the continental shelf benthos in the Arctic is from the shrinking of pack ice [118]. The consequence of this for the benthos is predicted to be a reduced carbon supply to the seafloor. If carbon is intercepted by zooplankton and the microbial loop, this would change the quality, timing, and source of carbon to the benthos [118]. This change could, in turn, alter species composition and reproductive cycles, thereby redistributing benthic biomass. Lower benthic biomass would presumably affect predators, including mammals and sea birds, favoring smaller predators such as fish [116].

The threats faced by the Arctic coastline are different from those on the Atlantic and Pacific coasts. Sparse human populations and an ice-covered ocean have helped to protect Arctic biodiversity from human activity in the past, but similar protection has not occurred on the Atlantic and Pacific coasts. Though effects of climate change may occur, particularly in transition regions such as northern Newfoundland [11], the most significant impact over broad scales is related to fishing effects on habitat [119], [120] and on pelagic [121] and benthic [122], [123] food webs. Decreased biomass and damage to animals with shells, such as bivalves and urchins, have also been observed [124]. A study of trawling effects on hard substrate fauna indicated relatively modest effects of trawling [125], in some cases as a result of rapid colonization and growth potential [126].

Though there is little doubt that the sedimentary infauna in Canadian waters is undersampled, it is difficult to know just how significant this undersampling actually is. Furthermore, using the approach of Griffiths et al. [127], it is possible to generate a crude estimate of this number. The ratio of European fishes to European polychaetes, both of which are assumed to be relatively well described, is 1.37. The equivalent ratio in Eastern Canada is 0.64. If we assume that the fishes of Eastern Canada waters are relatively well described (this is the most sampled province in Canada) and then make the large assumption that proportions of species within different phyla are similar in different areas of the world, this ratio suggests that only a little more than half of the polychaete species in Eastern Canada have been described. It is noteworthy, however, that in European and Canadian waters, the deep-water fauna is underestimated, potentially by an order of magnitude, at least for sedimentary infauna [128]. A major effort to sample all habitats from the intertidal zone to the deep sea, including hard-bottom substrata, needs to be undertaken if a true baseline of Canadian marine benthic biodiversity is to be established.

### Fish (total taxa 891–932)

Given the important roles played by marine fishes within Canada's culture, economy, and ecosystems, and considering information gained through their exploitation and management, knowledge of marine fish diversity can be said to be relatively well documented, yet continually expanding [129], [130], [131], [132]. The approximately 900 marine fish species reported from Canada's territorial waters among three oceans (Table 4) represent over 5% of all the fish species described in the OBIS global database [133]. In an international context, Canada is among only seven nations or large territories in which more than 80% of territorial marine fish species are estimated to have been discovered, based on spatial analyses of the completeness of the OBIS database [133]. However, within Canada, the Arctic Ocean has not been as thoroughly sampled as the Pacific and Atlantic [131], [133]. As a result, since the early 1960s, the number of known Arctic fish has nearly doubled. In addition to the 189 species reported for the Arctic (Table 4), some 83 additional species occur in adjacent non-Canadian waters and may yet be found to occur in Canada [129]. Some new species are likely to be found in the Arctic, particularly in deeper waters of the Atlantic and Pacific in groups such as the midwater fishes [133].

**Table 4:** Diversity and status of marine fishes in the three ocean provinces in Canada.

Province	Species (Families)	Current and potential threats	Committee on the Status of Endangered Wildlife in Canada^a^ (COSEWIC) marine fish species/population assessments					
			Endangered	Threatened	Special concern	Not at risk	Data deficient	Candidate species (April 2009)
Western Canada	371^b^ (99^c^)	Overexploitation; Bycatch; Potential future ocean warming	4	3	7	6	4	8
Canadian Arctic	189^d^ (48^d^)	Reduced sea ice leads to thermal habitat loss; Potential future overexploitation; Potential future bycatch	1	2	1	0	2	0
Eastern Canada	527^b^∼538^e^ (151^e^)	Overexploitation; Bycatch; Potential future ocean warming	6	6	5	1	1	7
Total^f^	891^b^∼932^g^ (193^g^)		11	11	13	7	7	15

a <http://www.cosewic.gc.ca>;

b [131];

c [269];

d [129];

e [132];

f Totals within COSEWIC columns include marine fish with populations in more than one ocean;

g ‘Native’ Canadian species and families from [270]).

Numbers of both Atlantic and Pacific species and families greatly exceed those reported from the Arctic (Table 4). These patterns reflect true spatial differences in total numbers of species among oceans, although comparisons of species richness between the Atlantic and the Pacific are strongly influenced by the relatively small area of Canada's Pacific coast [131], [134]. Even in relatively well sampled regions, such as the Atlantic Scotian Shelf, an area in which standardized trawl surveys have been conducted annually for decades, new records of fish species continue to be detected [135]. This pattern has led to examinations of additional physical correlates, including sampled area and depth range, as potential surrogates for fish species richness, in order to provide scientific advice related to fish conservation in the absence of exhaustive census data [135]. Despite challenges in enumerating fish diversity, it is critical to detect changes in the geographic distributions of fishes to quantify their dynamics in response to climate variability and exploitation. For example, latitudinal distributions and species-richness patterns of Atlantic fishes change from year to year in response to atmospherically influenced changes in ocean temperature [136]. Such positive relationships between water temperature and species richness portend future changes in response to increases or variability in ocean conditions. It was reported that in the Pacific [130], about 16% of species had their northern range limit, and 4% had their southern range limit, within Canadian waters; these boundaries may shift with future changing ocean conditions. Already, in the Bering Sea, a region that separates Canada's Pacific and Arctic ocean waters, decreases in ice cover and increases in water temperature on the continental shelf have led to northward shifts in marine fish distributions, increasing catch rates of some species, and increased species richness within the last 25 years [137]. Those results illustrate the value of repeated surveys in high-latitude marine ecosystems. Just as the Canadian Arctic has been important to the transfer of species among northern ocean basins in the geological past (particularly from the Pacific to Atlantic [134]), future decreases in the extent of Arctic sea ice are predicted to provide similar conditions that will facilitate the redistribution of fishes and invertebrates [138]. Given that Canada's Arctic waters are expected to be a zone of changing biodiversity in the coming years, yet remain relatively undersampled [129], [131], [133], increased monitoring will be required to detect future changes.

Although climate can certainly influence marine fish distributions and diversity, the majority of fishes that are of greatest concern (those considered top candidates for assessment by the Committee on the Status of Endangered Wildlife in Canada, COSEWIC) (Table 4) are listed because of the impacts of directed fisheries exploitation or bycatch. These patterns mirror wider assessments of North American and global marine fishes that are threatened mostly by exploitation, habitat loss, and pollution [139]. Whereas less than 2% of all Canadian marine fish species were formally assessed for extinction risk status, of those assessed, 53% were considered threatened [139]. Together with species that are considered to be “data deficient” (Table 4), species-specific patterns partly reflect the current logistical limits to knowledge of marine fish population dynamics beyond the most abundant and largest species. For species or populations that have been classified by COSEWIC as imperilled and recommended for protection, there are additional decisions at the federal government level that determine whether species are protected under existing Canadian species-at-risk legislation [140]. For marine fishes specifically, their listings between 2003 and 2006 greatly lagged species within other taxonomic groups (mostly terrestrial and freshwater species); only one of 11 marine fishes was listed, and this was a species not fished commercially [140]. In addition to changes in relatively shallow-water and low-latitude ecosystems, declines in fish abundance extend to Canada's deep-sea habitats [141], and the potential future establishment of commercial fishing in the Arctic will require assessments of both direct and indirect effects on Arctic ecosystems [142].

Sampling coverage within Canadian waters has recently accelerated as a direct result of the collection of new data and the amalgamation and dissemination of existing data by OBIS. McAllister [131], for example, called for increased systematic surveys of Canadian Arctic, mesopelagic, rocky bottom zones, and waters deeper than 500 m. Directed sampling has already increased the coverage of deep waters within Canada's east Arctic Baffin Bay and Davis Strait regions [143], and existing Canadian mesopelagic survey data are expected to be added to the OBIS database shortly (see www.marinebiodiversity.ca). Further, the increased profile for marine biodiversity in the last decade, as well as Census of Marine Life efforts to specifically target surveys within Arctic ecosystems, will undoubtedly contribute to increased spatial coverage. Currently, Atlantic data sources dominate Canadian marine fish representation in OBIS, partly as a result of data availability from nationally funded annual surveys in this region and partly as a result of differences in regional efforts to migrate existing data into OBIS. Current research also seeks a greater understanding of how and why patterns of marine fish diversity are changing. Therefore, in addition to the biological data contained in OBIS, it may be useful in the future to link specific samples in OBIS to concurrent oceanographic data (as are collected in many Canadian scientific fish surveys) or to match them to remotely sensed oceanographic data. Such linkages between biological, physical, and chemical databases would provide oceanographic and environmental contexts in which to evaluate changes in fish abundance and distribution for preserving Canada's marine ecosystems in the face of multiple stressors.

### Marine mammals (total 52 species)

Of the 125 extant marine mammal species worldwide, 52 occur in Canadian oceans, including representatives from all major taxa, except sirenians and river dolphins (Table S2). This total is five times higher than that reported previously [43]). Species diversity is highest in the eastern North Pacific (37 species), followed by the western North Atlantic (30 species), and the Arctic (24 species). In Eastern and Western Canada, species richness is among the highest reported worldwide for marine mammals, largely as a result of the diversity observed on the Scotian Shelf (Atlantic) and in Pacific coastal waters [144]. The wide distribution ranges of many marine mammals [144], which often include high-latitude feeding grounds and low-latitude breeding grounds, result in overlap in Canadian waters between temperate and more Arctic species, thus enhancing diversity. The long history of marine mammal exploitation, which provides indirect data and has stimulated scientific research efforts, is also likely to contribute to species discovery and high species richness in Eastern and Western Canada.

High primary productivity at 40 to 60 degrees north and south latitude [145] was proposed as a reason for the relatively high diversity of marine mammals in Canadian waters, although diversity is lower than expected in the North Atlantic based solely on primary productivity [144]. Other studies challenged this hypothesis as they found little correspondence between biodiversity and primary productivity in several species groups, including oceanic cetaceans [146], [147], [148]. It is unlikely that the lower-than-expected diversity results primarily from local extinction, given their small number (n = 3) in the western North Atlantic [144]. Instead, it might result from colder sea-surface temperatures in the North Atlantic than those observed at midlatitudes or along the Canadian Pacific coast. Species distribution and diversity appear to vary positively with sea-surface temperature in various taxa up to a certain temperature, above which a decline in diversity may occur in some species groups [146], [147], [148].

Nevertheless, it remains inarguable that commercial and, in some cases, subsistence exploitation have historically threatened several marine mammal species in Canadian oceans and elsewhere. The vast majority of the larger cetaceans were driven to near extinction worldwide by these past practices [149]. At least 33 of the 52 marine mammal species in Canada have been subjected to heavy exploitation, including 16 species that are still harvested today, either commercially or for subsistence, or simply because of the nuisance they cause to fisheries and other human activities or infrastructures (Table S2). Although some species (e.g., humpback whales) might be on their way to recovery, populations of seven of the eight larger whales are still considered at risk of extinction in Canada. In total, 22 species of marine mammals (42%) are at risk of extinction in Canada, including 9 of 30 populations in the Eastern Canada, 14 of 22 populations in the Arctic, and 14 of 37 populations in the Western Canada. This figure is higher than the overall proportion (36%) of marine mammal species at risk of extinction globally [144]. Although all at-risk populations are protected from hunting in Eastern Canada, 8 of the 14 species at risk in the Canadian Arctic and 6 of 14 populations at risk in Western Canada are still harvested for subsistence, or because they represent a nuisance.

Direct interactions with the world's fisheries also threaten marine mammals worldwide, including Canada. Each year fisheries probably kill hundreds of thousands of small cetaceans, and to a lesser extent pinnipeds and otters [144], [150]. In Canada, incidental capture of harbor porpoises and entanglement of some of the larger whales in fishing gear are of serious concern [151], [152], [153].

In addition to hunting and fisheries, habitat loss and degradation represent by far the main threats to marine mammals worldwide and may arise through ecological interactions with fisheries, climate change, or pollution [144], [154], [155]. In Canada, habitat loss or degradation through climate change is predicted to have dramatic consequences for strongly pagophilic Arctic species or for those with narrow ecological niches [156]; Ocean warming might cause an increase in diversity in Eastern and Western Canada by shifting northward the distribution of species currently found slightly south of Canada's borders [147].

Pollution effects on whales exposed to chemical contaminants, noise, and introduced pathogens and toxin-producing organisms, are also of growing concern worldwide and in Canada [144], [157], [158]. The best-documented cases of high pollutant accumulation in Canada are for species occupying high trophic positions, notably killer whales and harbor seals [159], [160], St. Lawrence beluga whales and harbor seals [161], [162], and polar bears [163]. Monitoring and predicting effects of these threats on Canadian biodiversity will require not only more extensive field-based observations but also new tools to track these changes remotely on a more global scale [154]. There is a need to better characterize the distribution of marine mammal species in Canada, particularly Arctic and deep-water species. However, it is doubtful that these censuses will lead to the discovery of new species, considering the long history of marine mammal exploitation and observation in Canadian waters. These survey efforts might instead enhance diversity by revealing range extensions of Arctic species to the south, or of temperate and subarctic species to the north.

## Discussion

### The known

Generally, most taxonomic groups contain higher numbers of species in southern marine areas than in the north [164], [165]. For example, only 189, or 21%, of Canada's marine fish are found in the Canadian Arctic (see Table 4). But this is not always true (Table 1), given that known phytoplankton species are markedly more species rich in the Canadian Arctic (1,002 species, Table 1) than elsewhere. Crustaceans are also more diverse in the Canadian Arctic than in Eastern and Western Canada (Tables 5, 6, and 7). Further, Western Canada is generally more species rich than Eastern Canada, even though less sampling effort has been expended in the former area (e.g., benthic infauna, Table 3). The west coast of Canada has one of the richest seaweed floras in the world (650 species; Table 2). Rhodophyta are well represented in Western Canada with 380 taxa, which is almost 3 times higher than in Eastern Canada and and 5.8 times higher than in the Canadian Arctic (Tables 5, 6, and 7). Some other taxonomic groups such Pheaophyta and Chlorophyta are nearly equal in species number among the three oceans.

**Table 5:** Taxonomic classification of taxa reported in Canadian Arctic.

Taxonomic group	No. taxa	State of knowledge	No. introduced species^2^	No. experts	No. ID guides
Domain Archaea	50–5000	1	ND	0	0
Domain Bacteria (including Cyanobacteria)	5004–50004	1	ND	0	0
Domain Eukarya					
Other Eukarya (5 phyla)	50–500	1	ND	2–3	0
Kingdom Chromista					
Phaeophyta	134	5	2?	∼10	1
Chromobiota (phyto)	774	2	ND	<5	2
Kingdom Plantae					
Chlorophyta	132	3	?	∼10	2+1
Rhodophyta	66	4	?	∼10	1
Angiospermae (not included in our analysis)	ND	ND	ND	ND	ND
Kingdom Protoctista (Protozoa)					
Dinomastigota (Dinoflagellata)	301	3	ND	<5	2
Foraminifera	ND	ND	ND	ND	ND
Unclassified Prototista	41	2	ND	<5	2
Unclassified choanoflagellates	30	2	ND	<5	2
Kingdom Animalia					
Porifera	4	2	ND	1	0
Cnidaria	47	3	ND	2	3
Platyhelminthes	1	1	ND	ND	0
Mollusca	156	3	ND	3	1
Annelida	324	3	ND	1	2
Crustacea	722	3	ND	3	9
Bryozoa/Ectoprocta	3	2	ND	ND	2
Echinodermata	35	3	ND	1	3
Urochordata (Tunicata)	3	2	ND	2	1
Other invertebrates	52	2	ND	2	1
Vertebrata (Pisces)	189	4	0	∼5	5
Marine mammals	24	4	0	15–20	4–5
SUBTOTAL	3038^1^				
TOTAL REGIONAL DIVERSITY	8142–58547				

**Notes:** The taxonomic classification of phytoplankton, zooplankton species reported in Canadian Arctic, including the Hudson Bay system (Hudson Bay, Hudson Strait, and Foxe Basin). The benthic taxa are only the infaunal species.

1 Subtotal before the domains Bacteria, Eukarya, Archaea.

2 The total number of introduced species in the three Canadian oceans is approximately 112. We know this is an incomplete count that needs to be updated (A Locke, JM Hanson, and JL Martin, manuscript in preparation).

**Table 6:** Taxonomic classification of taxa reported in Eastern Canada.

Taxonomic group	No. taxa	State of knowledge	No. introduced species^2^	No. experts	No. ID guides
Domain Archaea	50–5000	1	ND	0	0
Domain Bacteria (including Cyanobacteria)	5000–50000	1	ND	0	0
Domain Eukarya					
Other Eukarya (5 phyla)	50–500	1	ND	2–3	0
Kingdom Chromista					
Phaeophyta	120	5	1	∼10	1
Chromobiota (phytoplankton)	333	4	ND	<5	2
Kingdom Plantae					
Chlorophyta	121	3–5	1	∼10	3
Rhodophyta	130	5	2	>10	2
Angiospermae (not included in our analysis)	ND	ND	ND	ND	ND
Kingdom Protoctista (Protozoa)					
Dinomastigota (Dinoflagellata)	219	3	ND	<5	2
Foraminifera	ND	ND	ND	ND	ND
Unclassified Prototista	14	2	ND	<5	2
Unclassified choanoflagellates	29	2	ND	<5	2
Kingdom Animalia					
Porifera	6	2	ND	2	0
Cnidaria	97	4	ND	1+1(Ret)	2
Platyhelminthes	3	1	ND	ND	ND0
Mollusca	228	4	ND	2+1(Ret)	2
Annelida	439	3	ND	2	2+1
Crustacea	719	4	ND	9	8
Bryozoa/Ectoprocta	8	2	ND	0	2
Echinodermata	52	4	ND	ND	3
Urochordata (Tunicata)	ND	ND	ND	1	ND
Other invertebrates	72	1	ND	2	2
Vertebrata (Pisces)	538	5	1	∼10	3
Marine mammals	32	4	0	20–25	4–5
SUBTOTAL	3160^1^				
TOTAL REGIONAL DIVERSITY	8260–58660				

**Notes:** The taxonomic classification of phytoplankton, zooplankton species reported in Eastern Canada, including the St. Lawrence ecosystem. The benthic taxa are only the infaunal species.

1 Subtotal before the domains Bacteria, Eukarya, Archaea.

2 The total number of introduced species in the three Canadian oceans is approximately 112. We know this is an incomplete count that needs to be updated (A Locke, JM Hanson, and JL Martin, manuscript in preparation).

Ret = Retired.

**Table 7:** Taxonomic classification of species reported in Western Canada.

Taxonomic group	No. taxa	State of knowledge	No. introduced species^2^	No. experts	No. ID guides
Domain Archaea	50–5000	1	ND	0	0
Domain Bacteria (including Cyanobacteria)	5000–50000	1	ND	0	0
Domain Eukarya					
Other Eukarya (5 phyla)	50–500	1	ND	2–3	0
Kingdom Chromista					
Phaeophyta	134	5	2 ?	∼10	1
Chromobiota (phytoplankton)	355	4	ND	<5	2
Kingdom Plantae					
Chlorophyta	122	2–5	1 ?	∼10	3
Rhodophyta	380	5	ND	∼10	1
Angiospermae (not included in our analysis)	ND	ND	ND	ND	ND
Kingdom Protoctista (Protozoa)					
Dinomastigota (Dinoflagellata)	112	3	ND	<5	2
Foraminifera	ND	ND	ND	ND	ND
Unclassified Prototista	3	2	ND	<5	2
Unclassified choanoflagellates	ND	2	ND	<5	2
Kingdom Animalia					
Porifera	ND	ND	ND	3	0
Cnidaria	5	4	ND	1+1(Ret)	2
Platyhelminthes	2	1	ND	ND	0
Mollusca	188	3	ND	1 (Ret)	2
Annelida	364	3	ND	2	2
Crustacea	481	5	5?	3	7
Bryozoa/Ectoprocta	ND	2	ND	ND	2
Echinodermata	24	3	ND	1	3
Urochordata (Tunicata)	12	4	ND	1	1
Other invertebrates	46	4	ND	1	2
Vertebrata (Pisces)	371	5	2	∼10	4
Marine mammals	37	4	0	10–15	3–4
SUBTOTAL	2636^1^				
TOTAL REGIONAL DIVERSITY	7736–58136				

**Notes:** The benthic taxa are only the infaunal species.

1 Subtotal before the domains Bacteria, Eukarya, Archaea.

2 The total number of introduced species in the three Canadian oceans is approximately 112. We know this is an incomplete count that needs to be updated (A Locke, JM Hanson, and JL Martin, manuscript in preparation).

Ret = Retired.

Not surprisingly, the best-known groups of organisms are those that are relatively easily sampled (the macroalgae and presumably other intertidal to shallow-water fauna), those that are of greatest economic interest (the fishes), and those that are large and charismatic (marine mammals).

Macroalgae species (Rhodophyta, Chlorophyta, and Phaeophyta macroalgae) are generally taxonomically well known; about 830 species have been described from the region. There are approximately 900 species of fishes known, which probably represent more than 80% of those that occur in Canadian waters. Finally, Canadian waters include 52 species of marine mammals, which represent 44%, at least seasonally, of the marine mammals on the planet [166]. For most other groups of organisms, the proportion of unknown species is sufficiently large that extrapolation of a total number is difficult.

In a historical inventory of marine invertebrate taxa (intertidal, benthic, pelagic, parasitic) in the Haida Gwaii (Queen Charlotte Islands) region of Western Canada, a marine species accumulation curve was calculated using sampling data from the first record in 1878 to 2000 [167]. The curve accumulates a total of 2,276 species. This once more shows that Canadian marine invertebrate biodiversity is underestimated in the present study, as the total number of macroinvertebrate taxa enumerated in our study for the infauna and zooplankton taxa for the west coast of Canada is comparatively low with 1,122 taxa (Table 7). Differences between these two numbers are explained in part by the inclusion of organisms from hard-bottom habitat and parasitic species in the former study [167] and in part by the fact that the authors of that study worked from species inventories rather than raw data, as was done in the present study. This type of calculation may also overestimate total known species because a very careful review is needed by a wide range of taxonomic experts to ensure the validity and uniqueness of all taxa. This review will be far easier once the World Register of Marine Species (WoRMS) completes its global list of known of marine taxa.

Noting all of these caveats, the minimum number of taxa in the three Canadian oceans is currently between 15,988 and 61,148. This range is quite high even without sampling many areas of Canada and in view of our known underestimation of the taxa in Canadian oceans.

### The unknown

**Figure 5:**
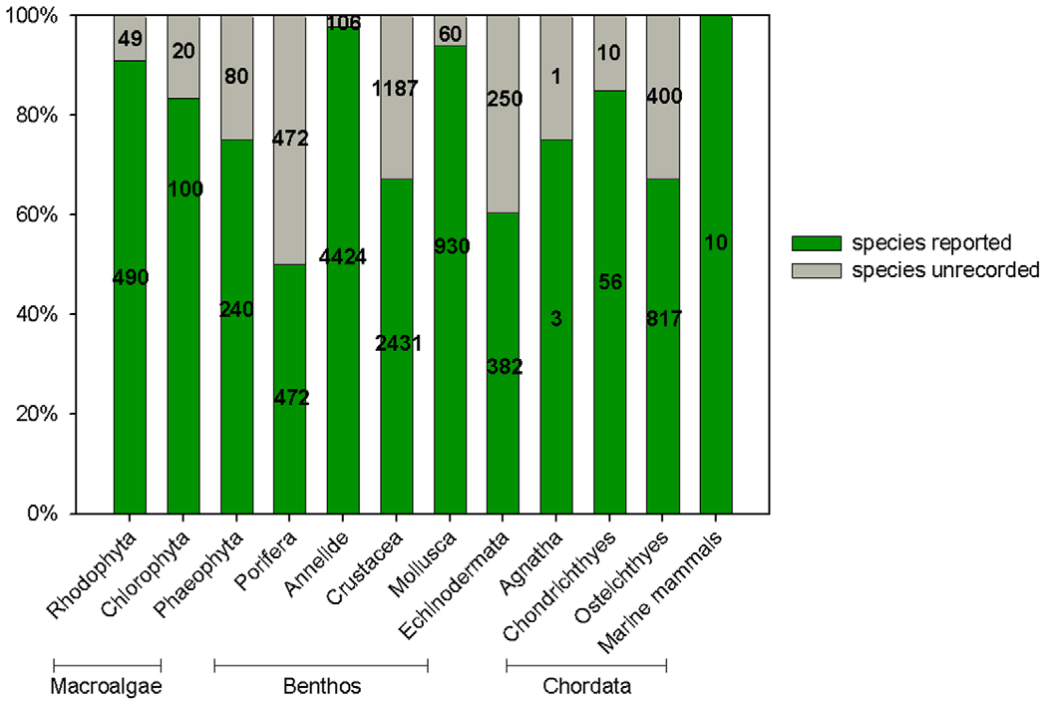
Percent contributions of reported (green) and unrecorded (gray) total numbers of species. Data are limited to sampling within Canada's 200-nautical-mile limit. This compilation has been produced from the data listed in Appendix 1 of Mosquin et al. [39].

Figure 5 shows data adapted from a summary table presented by Mosquin et al. [43] in their 1995 review of taxonomic diversity in Canada. We have chosen a few phyla and subdivisions of marine organisms to illustrate the number of species reported, versus those unrecorded in the literature at that time. The term “unrecorded” refers to the estimated gaps in our knowledge from the numbers of undescribed species or as yet unrecorded species in each taxonomic group. The information reported by Mosquin et al. [43] suggested that only 48% of marine species in Canada have been scientifically named and classified. Clearly, there is room for training new generations of taxonomists!

There are significant disparities in knowledge and status of taxonomic inventory across taxonomic groups. Even for most of the named marine species, ecological and life history information, as well as information on geographic distribution, is sparse. Grid-based biological surveys would provide the basis for sound distribution maps that are currently lacking for many species. In general, larger organisms, such as Chordata are represented by fewer taxa in Canada, and most are known (with the possible exception of a small proportion of Osteichthyes). However, even though there are relatively few marine mammal species in Canada, there is a major discrepancy between the total of 10 species listed [43] and the 52 species we have included (Table S2). This difference highlights the critical need to establish baseline knowledge of Canadian marine biodiversity. Considering how comparatively well known marine mammals are relative to most other groups, the inferred gaps in knowledge are particularly disconcerting when attempting to estimate the diversity of smaller organisms in poorly sampled taxonomic groups, such as benthic and pelagic invertebrates, phytoplankton, and microbes.

In addition to the disparity in taxonomic effort across different phyla, there is also strong habitat dependence with respect to species inventory; shallow, nearshore environments are much better sampled than deep-sea sediments. Deep-sea sediments represent the largest ecosystem type on Earth in area. The benthic organisms in and on sediments represent the largest proportion of unrecorded or undescribed metazoan diversity in Canadian waters. Indeed, the data from Mosquin et al. [43] in Figure 5 show that although the benthos, which encompasses 8,639 species, represents the largest group of described marine species, there are an additional 2,075 species that have been collected but remain unrecorded. This gap becomes even more striking when considering the vast extent of the deep-sea environment and the small amount that has been sampled.

These examples highlight the substantial gaps in current taxonomic knowledge and the need for better information to guide future conservation measures in marine ecosystems.

### Taxonomic challenges in Canadian marine research

The overall state of taxonomic effort in Canada has shown a serious decline over the past two to three decades. Reports produced in the mid-1990s suggested an impending crisis [168], [169], [170] and, as in many other disciplines in Canada, the number of taxonomists and systematists specializing in marine taxa has dropped at an alarming rate. A comparison of results from a 1996 survey [171] of marine taxonomists and systematists in Canada with those from an extensive revision carried out in 2004 [172] suggests attrition due to retirement as a major cause of this decline. A similar decline is observed in Europe [173]. Vacated positions in universities and government laboratories have not been filled by traditional taxonomists. While the number of respondents to the 2004 survey is significantly greater, the vast majority declared themselves as unavailable to do taxonomic work. Few who received formal taxonomic training actually work in a field where they can apply their taxonomic expertise in the exploration of biodiversity. Of those who are available, most have not received formal taxonomic training and may be best described as “parataxonomists,” in the broadest sense of the term. Tables 5, 6, and 7 showed clearly that the number of experts correlates with the size of the organisms studied. Marine mammals have 10 to 25 experts nationally, depending of the ocean province, pisces have more than 5 experts, and macroalgae have 10 experts. All other taxonomic groups have fewer than 5 experts or none (see Bryozoa, Archaea, Bacteria). For many phyla, expertise is often limited to a subset of families, with no capacity in other groups.

With increasing research emphasis on community ecology approaches and economically important species, and with decreasing funding for baseline taxonomic surveys and traditional taxonomy, very few traditional marine taxonomists have been trained in recent decades. Consequently, few taxonomic revisions and new descriptions of Canadian marine taxa have been published. To highlight this fact, a new species of polycheate in Canada was recently described [174], [175] by a Mexican taxonomist, because there was nobody in Canada available to take on the task. Population and community ecologists (who often have no choice but to use old and outdated taxonomic information to assign names to their specimens) have now become the parataxonomists responsible for training new parataxonomists. In this context, we expect a diminishing capacity to assign the correct taxonomic terms to marine species, and increasingly inaccurate taxonomy from one generation to the next. For instance, issues such as the *cosmopolitan species syndrome*, where species similar in appearance are given the same name based on the first-described taxon (often from the Old World literature), are perpetuated as a result of the limited recent taxonomic work and the reduced capacity of parataxonomists to distinguish subtle differences between sibling species. These issues necessitate care in analyses of merged databases where taxonomic precision and accuracy may be very unven [176].

Canada's marine taxonomic challenge is certainly exacerbated by the fact that a relatively small total scientific community is distributed across a large geographic area that borders on three of the world's oceans and has the longest national coastline in the world. Not surprisingly, for historical and geographic reasons, there is much better taxonomic coverage of the northwest Atlantic region than the west coast and particularly Canadian Arctic waters. These differences are reflected both in the number of preserved collections in museums and in taxonomic publications. This imbalance emphasizes the need to ensure the preservation of recently collected material and voucher (identified) specimens, especially if we are to retain the capacity to confirm species identifications at a later date. Unfortunately, with limited taxonomic research capacity in the world [177], even in Canadian museums, the wealth of knowledge contained in old and recently collected material will remain unavailable. This challenge was emphasized by the White Point Workshop on Marine Biodiversity in Canada [178], [179] in 2002, which recommended support for research programs with taxonomic inventories and support for collection-holding infrastructure. An official Survey of Taxonomic Expertise in Canada was undertaken in February 2010 by the Council of Canadian Academies' Expert Panel on Biodiversity Science, and results will be known later during 2010.

Fortunately, the rapid development of genetic approaches for identification of species, such as the Barcode of Life (University of Guelph, Ontario, Canada), has increased interest in taxonomy and systematics of marine taxa in Canada. Radulovici et al. [180] reviewed the utility of this method for marine organisms. These nontraditional approaches are encouraging but cannot yet take advantage of museum-preserved material. Taxonomic experts cannot always validate their results with this approach, thus limiting its utility. Mosquin et al. [43] projected that about 34% of Canadian marine invertebrates remain unreported (ranging from 8.1% for Mollusca to 49.5% for Porifera). Hence, there is no doubt that new approaches are needed to discover these estimated at least 3,500 unreported marine invertebrate species. Indeed, given the new genetic tools, it is likely that this number will increase significantly in the future. Greater investments will be needed to address these challenges. The traditional taxonomy and the molecular taxonomy need to be integrated together to describe what it is left to describe and not one method to the detriment of the other method [177]. Taxonomy must be seen as more than a descriptive exercise but as a fundamental tool of discovery, conservation, and management.

As an example, Saunders [181] and Robba et al. [182] evaluated barcoding for a range of red algal taxa from all three oceans. *Porphyra* provides a case study for cryptic speciation and the importance of wide-ranging geographic sampling to determine both evolutionary divergence and species' distributions [183]. Molecular sampling of hundreds of populations of 22 named species from California to Alaska revealed one to many populations of five clades that merit species rank. That most of the described and undescribed entities have been collected from at least one location within the west coast waters included in this review suggests that they are more widely distributed within the region.

The Barcode of Life is one of several Census of Marine Life projects with significant activity in Canada. The Future of Marine Animal Populations (FMAP) program is led from Dalhousie University and has provided many new insights into trends in fisheries, global patterns in biodiversity, and the movements of animals in the oceans [184]. The Pacific Ocean Shelf Tracking (POST) project is led from the Vancouver Aquarium and has provided new insight into movements of Pacific salmon species, sturgeon, and other species along the North Pacific coastline [185]. Canadian scientists have also been involved in other Census projects that have not focused on Canadian territorial waters, though the Arctic Ocean Diversity (ArcOD) project [186] has sampled widely in the Arctic and the Natural Geography of Inshore Areas (NaGISA) has included sampling sites in Atlantic Canada [187]. None of these projects has engaged in broad-scale species inventory, though the Gulf of Maine Area (GoMA) project has assembled species lists for that region and worked closely with the Canadian node of the Ocean Biogeographic Information System (OBIS) program at Bedford Institute of Oceanography, which has assembled extensive datasets produced by Fisheries and Oceans Canada over several decades [188].

The small size of the Canadian marine science community has the advantage of allowing a relatively closely knit group with the potential to work together nationally to address key issues with respect to marine biodiversity. One outgrowth of the Census of Marine Life has been the establishment of a national research program funded by the Natural Sciences and Engineering Research Council of Canada that partners biodiversity researchers from 15 Canadian universities, with researchers and managers at Fisheries and Oceans Canada, the federal agency charged with ocean management and policy in Canada. This program also partners with seven other government laboratories. The Canadian Healthy Oceans Network (CHONe) is a five-year program that will address some of the objectives of the Census beyond 2010, as the program extends until 2013 and beyond through collaborations established during the lifetime of the network. The CHONe will foster projects that include establishing biodiversity baselines in poorly sampled areas, as well as projects on ecosystem function and connectivity, and allows for open access to data from the network through OBIS and other databases. New species will be a challenge, and require the involvement of taxonomic experts around the world. The network will help guide the Canadian marine biodiversity community to ensure that all data collected in the future are entered into widely accessible databases that will remain available beyond the lifetime of any individual project. The challenges to achieving this goal are substantial. Particular programs may have specific and unique data needs, making standardization difficult. The concept of open access to data is still new, and there are few mechanisms in place to assist data rescue and make available old hard-copy datasets through OBIS or other platforms. Nonetheless, the utility of global-scale analysis [133] is compelling, and more information is always better than less.

Canadian academic and government researchers are acutely aware of the ongoing threats to marine biodiversity due to habitat destruction, overfishing, and pollution, and there is new concern over the possible impacts of climate change, ocean acidification, and invasive species. Some of these effects may enhance biodiversity, though most are expected to reduce it. All will contribute to changing biodiversity. Understanding the long-term ramifications of those changes from a human social perspective and in the context of ecosystem services and health remains a challenge and an important focal point for research in the coming years. In light of the difficulties encountered in compiling biogeographic and species syntheses for Canada's three oceans, as described in this review, we hope that work presented here will pave the way for future syntheses that might be begin with expert taxonomic monographs that update and amalgamate knowledge for different taxa, and then progress to integrative analyses across taxa, habitats, and oceans. These syntheses will be of great use to scientists and organizations dedicated to understanding and protecting the marine environment.

## Supporting Information

Text S1 Territorial sea data is from L. Pruett and J. Cimino, unpublished data, Global Maritime Boundaries Database (GMBD), Veridian - MRJ Technology Solutions, (Fairfax, Virginia, January, 2000) (excluding Caspian sea and 2,867,050 km2 of disputed territorial). Territorial Sea is defined under the United Nations Convention on the Law of the Sea (UNCLOS) as the 12-nautical mile zone from the baseline or low-water line along the coast. The coastal State's sovereignty extends to the territorial sea, including its sea-bed, subsoil, and air space above it. Foreign vessels are allowed “innocent passage” through those waters. Even though the established limit for a territorial sea is 12 nautical miles, some countries claim larger areas. Territorial seas with overlapping claims from different countries are shown separately as disputed territorial seas. UNCLOS is an international agreement that sets conditions and limits on the use and exploitation of the oceans. This Convention also sets the rules for the maritime jurisdictional boundaries of the different member states. The UNCLOS was opened for signature on 10 December 1982 in Montego Bay, Jamaica, and it entered into force on 16 November 1994. As of January 2000, there are 132 countries that have ratified UNCLOS. Given the uncertainties surrounding much of the delimitation of the territorial seas, these figures should be used with caution. Please refer to the original source for further information on the variables and collection methodologies or to the following Web site: http://earthtrends.wri.org/. For more information in UNCLOS please refer to the United Nations Web page at: http://www.un.org/Depts/los/index.htm.
(0.03 MB DOC)

Text S2 Coastal length data are based on the World Vector Shoreline, United States Defense Mapping Agency, 1989. Figures were calculated by L. Pruett and J. Cimino, unpublished data, Global Maritime Boundaries Database (GMBD), Veridian - MRJ Technology Solutions, (Fairfax, Virginia, January, 2000).
(0.02 MB DOC)

Table S1 Numbers of marine zooplankton by family and species.
(0.11 MB DOC)

Table S2 Occurrence, status, and demographic trends of species and populations of marine mammals (Eastern Canada, Canadian Arctic, and Western Canada provinces).
(0.10 MB DOC)

Alternative Language Abstract S1 Abstract in French - Résumé en français. French translation of the abstract by Philippe Archambault.
(0.02 MB DOC)

## References

[1] Devine JA, Baker KD, Haedrich RL (2006). Deep-sea fishes qualify as endangered.. Nature.

[2] Myers RA, Hutchings JA, Barrowman NJ (1997). Why do fish stocks collapse? The example of cod in Atlantic Canada.. Ecol Appl.

[3] Smetacek V, Nicol S (2005). Polar ocean ecosystems in a changing world.. Nature.

[4] http://www.un.org/Depts/los/consultative_process/documents/7_mageau.pdf

[5] Airoldi L, Beck WM (2007). Loss, status and trends for coastal marine habitats of Europe.. Oceanogr Mar Biol Annu Rev.

[6] Hutchings JA (2000). Collapse and recovery of marine fishes.. Nature.

[7] Rossong MA, Williams PJ, Comeau M, Mitchell SC, Apaloo J (2006). Agonistic interactions between the invasive green crab, *Carcinus maenas* (Linnaeus) and juvenile American lobster, *Homarus americanus* (Milne Edwards).. J Exp Mar Biol Ecol.

[8] Dayton PK, Thrush SF, Agardy MT, Hofman RJ (1995). Environmental effects of marine fishing.. Aquat Conserv.

[9] Kenchington ELR, Prena J, Gilkinson KD, Gordon DCJ, MacIsaac K (2001). Effects of experimental otter trawling on the macrofauna of a sandy bottom ecosystem on the Grand Banks of Newfoundland.. Can J Fish Aquat Sci.

[10] Frank KT, Petrie B, Choi JS, Leggett WC (2005). Trophic cascades in a formerly cod-dominated ecosystem.. Science.

[11] Quijón PA, Snelgrove PVR (2005). Polychaete assemblages of a sub-arctic Newfoundland fjord: habitat, distribution, and identification.. Polar Biol.

[12] Coakley JP, Poulton DJ (1993). Source-related classification of St-Lawrence estuary sediments based on spatial-distribution of adsorbed contaminants.. Estuaries.

[13] Grime JP (1997). Ecology - Biodiversity and ecosystem function: The debate deepens.. Science.

[14] Hooper DU, Chapin IFS, Ewel A, Inchausti HP, Lavorel S (2005). Effects of biodiversity on ecosystems functioning: a consensus of current knowledge.. Ecol Monogr.

[15] Loreau M (2001). Biodiversity and ecosystem functionning: recent theorical advances.. Oikos.

[16] Ormond RFG (1996). Marine biodiversity: Causes and consequences.. J Mar Biol Assoc UK.

[17] Snelgrove PVR, Austin MC, Hawkins SJ, Iliffe TM, Kneib RT, Whitlatch RB, Levin LA, Weslawski JM, Garey JR, Wall DH (2004). Vulnerability of Marine Sedimentary Ecosystem Services to Human Activities.. Sustaining biodiversity and ecosystem services in soils and sediments.

[18] Roberts CM, Hawkins JP (1999). Extinction risk in the sea.. Trends Ecol Evol.

[19] Bouchet P, Duarte CM (2006). The magnitude of marine biodiversity.. The Exploration of Marine Biodiversity: Scientific and Technological Challenges.

[20] Snelgrove PVR (1998). The biodiversity of macrofaunal organisms in marine sediments.. Biodivers Conserv.

[21] Spalding MD, Fox HE, Halpern BS, McManus MA, Molnar J (2007). Marine ecoregions of the world: A bioregionalization of coastal and shelf areas.. Bioscience.

[22] Stroeve JC, Serreze MC, Fetterer F, Arbetter T, Meier W (2005). Tracking the Arctic's shrinking ice cover: Another extreme September minimum in 2004.. Geophys Res Lett.

[23] Reddy MPM (2001). Descriptive physical oceanography.

[24] Carmack EC, Walker WOJ (1990). Large-scale physical oceanography of polar oceans.. Polar oceanography Part A Physical science.

[25] Cusson M, Archambault P, Aitken A (2007). Biodiversity of benthic assemblages on the Arctic continental shelf: historical data from Canada.. Mar Ecol Prog Ser.

[26] Witman JD, Cusson M, Archambault P, Pershing AJ, Mieszkowska N (2008). The relation between productivity and species diversity in temperate-arctic marine ecosystems.. Ecology.

[27] McLaughlin F, Carmack EC, Ingram RG, Williams W, Michel C, Robinson AR, Brink KH (2004). Oceanography of the Northwest Passage.. The Sea Vol 14: The Global Coastal Ocean, Interdisciplinary Regional Studies and Syntheses.

[28] Collin AE, Dunbar MJ (1964). Physical Oceanography in Arctic Canada.. Oceanogr Mar Biol.

[29] Sanderson BG, LeBlond PH (1984). The cross-channel flow at the entrance of Lancaster Sound.. Atmos Ocean.

[30] Straneo F, Saucier F, Dickson RR, Meincke J, Rhines P (2008). The Arctic-Subarctic exchange through Hudson strait.. Arctic–Subarctic Ocean Fluxes.

[31] Colbourne EB, de Young B, Narayanan S, Helbig J (1997). Comparison of hydrology and circulation on the Newfoundland Shelf during 1990–1993.. Can J Fish Aquat Sc.

[32] Fennel W, Gilbert D, Su J, Urban ERJ, Sundby B, Malanotte-Rizzoli P, Melillo JM (2009). Physical processes in a semi-enclosed marine systems.. Watersheds, bays, and bounded seas: The science and management of semi-enclosed marine systems. SCOPE 70.

[33] Liu K-K, Seitzinger S, Mayorga E, Harrison J, Ittekkot V, Urban ERJ, Sundby B, Malanotte-Rizzoli P, Melillo JM (2009). Fluxes of nutrients and selected organic polluants carried by rivers.. Watersheds, bays, and bounded seas: The science and management of semi-enclosed marine systems SCOPE 70.

[34] Saucier FJ, Roy F, Gilbert D, Pellerin P, Ritchie H (2003). Modeling the formation and circulation processes of water masses and sea ice in the Gulf of St. Lawrence, Canada.. J Geophys Res-Oceans.

[35] Gilbert D, Chabot D, Archambault P, Rondeau B, Hébert S (2007). Appauvrissement en oxygène dans les eaux profondes du Saint-Laurent marin.. Nat Can.

[36] Gilbert D, Sundby B, Gobeil C, Mucci A, Tremblay G-H (2005). A seventy-two-year record of diminishing deep-water oxygen in the St. Lawrence estuary: the northwest Atlantic connection.. Limnol Oceanogr.

[37] Loder JW, Petrie B, Gawarkiewicz G, Robinson AR, Brink KH (1998). The coastal ocean off northeastern North America: A large-scale view.. The Sea, vol. 11.

[38] Thurston H (1990). Tidal life, a natural history of the Bay of Fundy.

[39] Thomson RE (1981). Oceanography of the British Columbia coast.. Can Spec Publ Fish Aquat Sci.

[40] Thomson RE, Hickey BM, LeBlond PH, Beamish R, McFarlane G (1989). The Vancouver Island coastal current: Fisheries barrier and conduit.. Effects of ocean variability on recruitment and an evaluation of parameters used in stock assessment models. Can Spec Publ Fish Aquat Sci.

[41] Crawford WR, Cherniawsky JY, Cummins P (1999). Surface currents in British Columbia coastal waters: Comparison of observations and model predictions.. Atmos-Ocean.

[42] Crawford WR, Thomson RE (1991). Physical Oceanography of the Western Canadian Continental-Shelf.. Cont Shelf Res.

[43] Mosquin T, Whiting PG, McAllister DE (1995). Canada's biodiversity: The variety of life, its status, economic benefits, conservation costs and unmet needs.

[44] Tunniclife V, Fenger MA, Miller EH, Johnson JA, Williams EJ (1993). Biodiversity: the marine biota of British Columbia.. Our Living Legacy: Proceedings of a Symposium on Biological Diversity.

[45] Brunel P, Bossé L, Lamarche G (1998). Catalogue of the marine invertebrates of the Estuary and Gulf of Saint-Lawrence.

[46] Zeller DR, Froese R, Pauly D (2005). On losing and recovering fisheries and marine science data.. Mar Policy.

[47] Huntsman AG, J Hjort Dep Nav Servo Ottawa, editor. (1919). Some quantitative and qualitative plankton studies of the eastern Canadian plankton. 3. A special study of the Canadian chaetognaths, their distribution etc., in waters of the eastern coast.. Can Fish Exped 1914-15.

[48] Willey A (1929). Notes on the distribution of free-living Copepoda in Canadian waters. Part II. Some intertidal harpacticoids from St. Andrews, New Brunswick.. Contrib Can Biol Fish.

[49] Fraser CM (1937). Hydroids of the Pacific coast of Canada and the United States.

[50] Prefontaine G, Brunel P (1962). Liste d'invertebres marins recueillis dans l'estuaire du Saint-Laurent de 1929 à 1934.. Nat Can.

[51] Whiteaves IF (1901). Catalogue of the marine invertebrata of eastern Canada.. Geol Surv Can Publ.

[52] Brunel P (1970). Catalogue d'invertebres benthiques du golfe Saint-Laurent recueillis de 1951 à 1966 par la station de biologie marine de Grande-Riviere.. Trav Pêch Qué.

[53] Bousfiel EL (1960). Canadian Atlantic sea shells..

[54] Bosse L, Sainte-Marie B, Fournier J (1996). Les invertebres des fonds meubles et la biogeographie du fjord du Saguenay.. Rapp Tech Can Sci Halieut Aquat.

[55] Gabrielson PW, Scagel RF, Widdowson TB (1990). Keys to the benthic marine algae and seagrasses of British Columbia, Southeast Alaska, Washington and Oregon..

[56] Squires HJ (1990). Decapod crustacea of the Atlantic Coast of Canada.. Can Bull Fish Aquat Sci.

[57] Banse K, Hobson KD (1974). Benthic errantiate polychaetes of British Columbia and Washington.. Bull Fish Res Board Can.

[58] Brinkhurs RO, Baker HR (1979). A review of the marine Tubificidae (Oligochaeta) of North America.. Can J Zool.

[59] Lubinsk I (1980). Marine bivalve molluscs of the Canadian central and eastern Arctic: faunal composition and zoogeography.. Can Bull Fishs Aaquat Sc.

[60] Hart JFL (1982). Crabs and their relatives of British Columbia..

[61] Margolis L, Arai HP, Kennedy MJ (1989). Parasites of marine mammals.. Synopsis of the parasites of vertebrates of Canada.

[62] McDonald TE, Margolis L (1995). Synopsis of the parasites of the fishes of Canada: supplement (1978–1993).. Can Spec Pubi Fish Aquat Sci.

[63] Bano N, Hollibaugh JT (2002). Phylogenetic composition of bacterioplankton assemblages from the Arctic Ocean.. Appl Environ Microb.

[64] Galand PE, Casamayor EO, Kirchman DL, Potvin M, Lovejoy C (2009). Unique archaeal assemblages in the Arctic Ocean unveiled by massively parallel tag sequencing.. Isme J.

[65] Galand PE, Lovejoy C, Hamilton AK, Ingram RG, Pedneault E (2009). Archaeal diversity and a gene for ammonia oxidation are coupled to oceanic circulation.. Environ Microbiol.

[66] Galand PE, Lovejoy C, Pouliot J, Garneau ME, Vincent WF (2008). Microbial community diversity and heterotrophic production in a coastal Arctic ecosystem: A stamukhi lake and its source waters.. Limnol Oceanogr.

[67] Galand PE, Lovejoy C, Pouliot J, Vincent WF (2008). Heterogeneous archaeal communities in the particle-rich environment of an arctic shelf ecosystem.. J Mar Syst.

[68] Galand PE, Lovejoy C, Vincent WF (2006). Remarkably diverse and contrasting archaeal communities in a large arctic river and the coastal Arctic Ocean.. Aquat Microb Ecol.

[69] Garneau ME, Vincent WF, Alonso-Saez L, Gratton Y, Lovejoy C (2006). Prokaryotic community structure and heterotrophic production in a river-influenced coastal arctic ecosystem.. Aquat Microb Ecol.

[70] Lovejoy C, Massana R, Pedros-Alio C (2006). Diversity and distribution of marine microbial eukaryotes in the Arctic Ocean and adjacent seas.. Appl Environ Microb.

[71] Sogin ML, Morrison HG, Huber JA, Mark Welch D, Huse SM (2006). Microbial diversity in the deep sea and the underexplored “rare biosphere”.. P Natl Acad Sci USA.

[72] Wells LE, Deming JW (2003). Abundance of Bacteria, the Cytophaga-Flavobacterium cluster and Archaea in cold oligotrophic waters and nepheloid layers of the Northwest Passage, Canadian Archipelago.. Aquat Microb Ecol.

[73] Terrado R, Lovejoy C, Massana R, Vincent WF (2008). Microbial food web responses to light and nutrients beneath the coastal Arctic Ocean sea ice during the winter-spring transition.. J Mar Syst.

[74] Terrado R, Vincent WF, Lovejoy C (2009). Mesopelgaic protists: diversty and succession in a coastal Arctic ecosystem.. Aquat Microb Ecol.

[75] Hamilton AK, Lovejoy C, Galand PE, Ingram RG (2008). Water masses and biogeography of picoeukaryote assemblages in a cold hydrographically complex system.. Limnol Oceanogr.

[76] Lovejoy C, Price NM, Legendre L (2004). Role of nutrient supply and loss in controlling protist species dominance and microbial food-webs during spring blooms.. Aquat Microb Ecol.

[77] Massana R, Terrado R, Forn I, Lovejoy C, Pedros-Alio C (2006). Distribution and abundance of uncultured heterotrophic flagellates in the world oceans.. Environ Microbiol.

[78] Carmack EC (2007). The alpha/beta ocean distinction: A perspective on freshwater fluxes, convection, nutrients and productivity in high latitudes seas.. Deep-Sea Res Pt II.

[79] DeLong EF (2009). The microbial ocean from genomes to biomes.. Nature.

[80] Sieburth JM, Smetacek V, Lenz J (1978). Pelagic ecosystem structure: Heterotrophic compartments of the plankton and their relationship to plankton size fractions.. Limnol Oceanogr.

[81] Falkowski PG, Katz ME, Knoll AH, Quigg A, Raven JA (2004). The evolution of modern eukaryotic phytoplankton.. Science.

[82] Sournia A, Chrétiennot-Dinet M-J, Ricard M (1991). Marine phytoplankton: how many species in the world ocean?. J Plankton Res.

[83] Tett P, Barton ED (1995). Why are there about 5000 species of phytoplankton in the sea?. J Plankton Res.

[84] Adl SM, Simpson AGB, Farmer MA, Andersen RA, Anderson OR (2005). The new higher level classification of eukaryotes with emphasis on the taxonomy of protists.. J Eukaryot Microbiol.

[85] Simon N, Cras A-L, Foulon E, Lemée R (2009). Diversity and evolution of marine phytoplankton.. C R Biol.

[86] Brunel J (1962). Le phytoplancton de la Baie des Chaleurs.. Contributions du Ministère de la chasse et des pêcheries.

[87] Bérard-Therriault L, Poulin M, Bossé L (1999). Guide d'identification du phytoplankton marin de l'estuaire et du golfe Saint-Laurent incluant également certains protozoaires.. Publ Spec Can Sci Halieut Aquat.

[88] Grøntved J, Seidenfaden G (1938). The phytoplankton of the waters west of Greenland.. Medd Grønl.

[89] Hsiao SIC (1983). A checklist of marine phytoplankton and sea ice microalgae recorded from Arctic Canada.. Nova Hedwigia.

[90] Poulin M, Lundholm N, Bérard-Therriault L, Starr M, Gagnon R (2010). Morphological and phylogenetic comparisons of *Neodenticula seminae* (Bacillariophyta) populations between the Subarctic Pacific and the Gulf of St. Lawrence.. Eur J Phycol.

[91] Reid PC, Johns DG, Edwards M, Starr M, Poulin M (2003). A biological consequence of reducing Arctic ice cover: arrival of the Pacific diatom *Neodenticula seminae* in the North Atlantic for the first time in 800 000 years.. Glob Change Biol.

[92] Starr M, St-Amand L, Bérard-Therriault L (2002). State of phytoplankton in the Estuary and Gulf of St. Lawrence during 2001.. DFO Can Sci Advis Sec Res Doc.

[93] Lüning K (1990). Seaweeds – their environment, biogeography, and ecophysiology.

[94] Garbary DJ, South GR (1990). Evolutionary biogeography of the marine algae of the North Atlantic.

[95] Garbary DJ, John Wiley & Sons L, ed. (2001). Biogeography of Marine Algae.. Encyclopedia of Life Sciences.

[96] Norton TA, Melkonian M, Anderson RA (1996). Algal biodiversity.. Phycologia.

[97] Scagel RF, Gabrielson PW, Garbary DJ, Golden L, Hawkes MW (1993). A synopsis of the benthic marine algae of British Columbia southeast Alaska, Washington and Oregon.

[98] Sears JR (1998). NEAS keys to the benthic marine algae of the northeastern coast of North America from Long Island Sound to the Strait of Belle Isle.

[99] South GR (1984). A checklist of marine algae of eastern Canada, second revision.. Can J Bot.

[100] South GR, Tittley I (1986). A catalogue and distributional index of the benthic marine algae of the North Atlantic Ocean.

[101] Lee RKS (1980). A catalogue of the marine algae of the Canadian Arctic.. Nat Mus Can Publ Bot.

[102] Lindstrom S (2006). Biogeography of Alaskan seaweeds.. J Appl Phycol.

[103] Lindstrom S (2001). The Bering Strait connection: dispersal and speciation in boreal macroalgae.. J Biogeogr.

[104] Adey W, Lindstrom S, Hommersand M, Müller K (2008). The biogeographic origin of Arctic endemic seaweeds: a thermogeographic view.. J Phycol.

[105] Dunton K (1992). Arctic biogeography: the paradox of the marine benthic fauna and flora.. Trends Ecol Evol.

[106] Wilce RT, Garbary DJ, South GR (1990). Role of the Arctic Ocean as a bridge between the Atlantic and Pacific Oceans: fact and hypothesis.. Evolutionary Biogeography of the Marine Algae of the North Atlantic.

[107] Mackas DL, Thomson RE, Galbraith M (2001). Changes in the zooplankton community of the British Columbia continental margin, 1985–1999, and their covariation with oceanographic conditions.. Can J Fish Aquat Sci.

[108] Johnson WS, Allen DM (2005).

[109] Shih CT, Figueira AJ, Grainger EH (1971). A synopsis of Canadian marine zooplankton.. Fish Res Board Can Bull.

[110] Lavaniegos BE, Ohman MD (2003). Long-term changes in pelagic tunicates of the California Current.. Deep-Sea Res Pt II.

[111] Lavaniegos BE, Ohman MD (2007). Coherence of long-term variations of zooplankton in two sectors of the California Current System.. Prog Oceanogr.

[112] Mackas DL, Galbraith M (2002). Zooplankton community composition along the inner portion of Line P during the 1997–1998 El Nino event.. Prog Oceanogr.

[113] Springer AM, McRoy CP, Turco KR (1989). The paradox of pelagic food webs in the northern Bering Sea-II. Zooplankton communities.. Cont Shelf Res.

[114] Eleftheriou A, McIntyre A (2005). Methods for the study of marine benthos.

[115] Thomson DH (1982). Marine benthos in the Eastern Canadian high arctic: multivariate analyses of standing crop and community structure.. Arctic.

[116] Conlan K, Aitken A, Hendrycks E, McClelland C, Melling H (2008). Distribution patterns of Canadian Beaufort shelf macrobenthos.. J Mar Syst.

[117] Lapoussière A, Michel C, Gosselin M, Poulin M (2009). Spatial variability in organic material sinking export in the Hudson Bay system, Canada, during fall.. Cont Shelf Res.

[118] Piepenburg D (2005). Recent research on Arctic benthos: common notions need to be revised.. Polar Biol.

[119] Kaiser MJ, Edwards DB, Armstrong PJ, Radford K, Lough NEL (1998). Changes in megafaunal benthic communities in different habitats after trawling disturbance.. J Mar Sci.

[120] Sheppard C (2006). Trawling the sea bed.. Mar Pollut Bull.

[121] Fisher JAD, Frank KT (2002). Changes in finfish community structure associated with an offshore fishery closed area on the Scotian Shelf.. Mar Ecol Prog Ser.

[122] Quijon PA, Snelgrove PVR (2005). Predation regulation of sedimentary faunal structure: potential effects of a fishery-induced switch in predators in a Newfoundland sub-Arctic fjord.. Oecologia.

[123] Worm B, Myers RA (2003). Meta-analysis of cod-shrimp interactions reveals top-down control in oceanic food webs.. Ecology.

[124] Prena J, Schwinghamer P, Rowell TW, Gordon DCJ, Gilkinson K (1999). Experimental otter trawling on a sandy bottom ecosystem of the Grand Banks of Newfoundland: analysis of trawl bycatch and effects on epifauna.. Mar Ecol Prog Ser.

[125] Kenchington ELR, Gilkinson KD, MacIssaac KG, Bourbonnais-Boyce C, Kenchington TJ (2006). Effects of experimental otter trawling on benthic assemblages on Western Bank, northwest Atlantic Ocean.. J Sea Res.

[126] Henry L-A, Kenchington ELR, Kenchington TJ, MacIsaac KG, Bourbonnais-Boynce C (2006). Impacts of otter trawling on colonial epifaunal assemblages on a cobble bottom ecosystem on Western Bank (northwest Atlantic).. Mar Ecol Prog Ser.

[127] Griffiths CL, Robinson TB, Lange L, Mead A (2010). Marine Biodiversity in South Africa: an Evaluation of Current States of Knowledge.. PlosOne.

[128] Snelgrove PVR (1999). Getting to the bottom of marine biodiversity: sedimentary habitats.. Bioscience.

[129] Coad BW, Reist JD (2004). Annotated list of the Arctic marine fishes of Canada.. Can Manuscr Rep Fish Aquat Sci.

[130] Hart JL (1973). Pacific Fishes of Canada.. Fish Res Board Can Bull.

[131] McAllister DE (1990). A List of the Fishes of Canada.. Syllogeus - Natl Mus Nat Sci.

[132] Scott WB, Scott MG (1988). Atlantic Fishes of Canada.. Can Bull Fish Aquat Sci.

[133] Mora C, Tittensor DP, Myers RA (2008). The completeness of taxonomic inventories for describing the global diversity and distribution of marine fishes.. P R Soc B.

[134] Briggs JC (1970). A faunal history of the North Atlantic Ocean.. Syst Zool.

[135] Shackell NL, Frank KT (2003). Marine fish diversity on the Scotian Shelf, Canada.. Aqua Cons Mar Freshw Ecosys.

[136] Fisher JAD, Frank KT, Petrie B, Leggett WC, Shackell NL (2008). Temporal dynamics within a contemporary latitudinal diversity gradient.. Ecol Lett.

[137] Meuter FJ, Litzow ME (2008). Sea ice retreat alters the biogeography of the Bering Sea continental shelf.. Ecol Appl.

[138] Vermeij GJ, Roopnarine PD (2008). The coming Arctic invasion.. Science.

[139] Reynolds JD, Dulvy NK, Goodwin NB, Hutchings JA (2005). Biology of extinction risk in marine fishes.. P R Soc B.

[140] Mooers AØ, Prugh LR, Festa-Bianchet M, Hutchings JA (2007). Biases in legal listing under Canadian endangered species legislation.. Conserv Biol.

[141] Baker KD, Devine JA, Haedrich RL (2009). Deep-sea fishes in Canada's Atlantic: population declines and predicted recovery times.. Environ Biol Fishes.

[142] Power G, Reynolds J (1997). A review of fish ecology in Arctic North America.. Fish Ecology in Arctic North America Bethesda: Am Fish Soc Symp.

[143] Jørgensen OA, Hvingel C, Møller PR, Treble MA (2005). Identification and mapping of bottom fish assemblages in Davis Strait and southern Baffin Bay.. Can J Fish Aquat Sci.

[144] Schipper J, Chanson JS, Chiozza F, Cox NA, Hoffmann M (2008). The status of the World's land and marine mammals: diversity, threat, and knowledge.. Science.

[145] Field CB, Behrenfeld MJ, Randerson JT, Falkowski PG (1998). Primary production of the biosphere: integrating terrestrial and oceanic components.. Science.

[146] Rutherford S, D'Hondt S, Prell W (1999). Environmental controls on the geographic distribution of zooplankton diversity.. Nature.

[147] Whitehead H, McGill B, Worm B (2008). Diversity of deep-water cetaceans in relation to temperature: implications for ocean warming.. Ecol Lett.

[148] Worm B, Sandow M, Oschlies A, Lotze HK, Myers RA (2005). Global patterns of predator diversity in the open oceans.. Science.

[149] Perry SL, DeMaster DP, Silber GK (1999). The great whales: history and status of six species listed as Endangered under the U.S. Endangered Species Act of 1973.. Mar Fish Rev.

[150] Read AJ (2008). The looming crisis: interactions between marine mammals and fisheries.. J Mammal.

[151] COSEWIC (2003). COSEWIC assessment and update status reports on the harbour porpoise *Phocoena phocoena* (Pacific Ocean population) in Canada.

[152] COSEWIC (2003). COSEWIC assessment and update status report on the North Atlantic right whale *Eubalaena glacialis* in Canada.

[153] COSEWIC (2006). COSEWIC assessment and update status reports on the harbour porpoise *Phocoena phocoena* (Northwest Atlantic population) in Canada.

[154] Buchanan GM, Nelson A, Mayaux P, Hartley A, Donald PF (2008). Delivering a global, terrestrial, biodiversity observation system through remote sensing.. Conserv Biol.

[155] Harwood J (2001). Marine mammals and their environment in the twenty-first century.. J Mammal.

[156] Kovacs KM, Lydersen C (2008). Climate change impacts on seals and whales in the North Atlantic Arctic and adjacent shelf seas.. Sci Prog.

[157] Geraci J, Harwood J, Lounsbury A, Twiss JR, Reeves R (1999). Marine mammal die-offs: causes, investigations and issues.. Conservation and management of marine mammals.

[158] Tyack PL (2008). Implications for marine mammals of large-scale changes in the marine acoustic environment.. J Mammal.

[159] Ross PS, Ellis GM, Ikonumou MG, Barrett-Lennard LG, Addison RE (2000). High PCB concentrations in free-ranging Pacific killer whales, *Orcinus orca*: effects of age, sec and dietary preference.. Mar Pollut Bull.

[160] Ross PS, Jeffries SJ, Yunker MB, Addison RE, Ikonumou MG (2004). Harbour seals (*Phoca vitulina*) in British Columbia, Canada, and Washington State, USA, reveal a combination of local and global polychlorinated byphenyl, dioxin and furan signals.. Environ Toxicol Chem.

[161] Bernt KE, Hammill MO, Lebeuf M, Kovacs KM (1999). Levels and patterns of PCBs and OC pesticides in harbour and grey seals from the St Lawrence Estuary, Canada.. Sci Total Environ.

[162] Lebeuf M, Gouteux B, Measures L, Trottier S (2004). Levels and temporal trends (1988–1999) of polybrominated diphenyl ethers in beluga whales (*Delphinapterus leucas*) from the St. Lawrence Estuary, Canada.. Envir Sci Technol.

[163] COSEWIC (2008). COSEWIC assessment and update status report on the polar bear *Ursus maritimus* in Canada.

[164] Gray JS, Poore GCB, Ugland KI, Wilson RS, Olsgard F (1997). Coastal and deep-sea benthic diversities compared.. Mar Ecol Prog Ser.

[165] Rombouts I, Beaugrand G, Ibanez F, Gasparini S, Chiba S (2009). Global latitudinal variations in marine copepod diversity and environmental factors.. P R Soc B.

[166] Jefferson TA, Leatherwood S, Webber MA (1993). FAO species identification guide.

[167] Sloan NA, Bartier PM (2009). Historic marine invertebrate species inventory: case study of a science baseline towards establishing a marine conservation area.. Aquat Conserv.

[168] Environment Canada (1995). Canadian Biodiversity Strategy - Canada's Response to the Convention on Biological Diversity. Biodiversity Convention Office.

[169] Federal Biosystematics Group (1995). Systematics - an impending crisis.

[170] Task Force on Canadian Biosystematics (1993). Task Force on Canadian Biosystematics - Report One..

[171] Canadian Museum of Nature (1996). Data Bank of Canadian Systematists and Taxonomists.Manuscript and report..

[172] Canadian Museum of Nature (2004). Canadian Marine Taxonomists..

[173] Costello MJ, Bouchet P, Emblow CS, Legakis A (2006). European marine biodiversity inventory and taxonomic resources: state of the art and gaps in knowledge.. Mar Ecol Prog Ser.

[174] Tovar-Hernandez MA (2007). Revision of Chone Krøyer, 1856 (Polychaeta: Sabellidae) from North America and descriptions of four new species.. J Nat Hist.

[175] Tovar-Hernandez MA (2007). On some species of Chone Krøyer, 1856 (Polychaeta: Sabellidae) from world-wide localities.. Zootaxa.

[176] Robertson MR (2008). Global biogeographical data bases on marine fishes: caveat emptor.. Diversity Distrib.

[177] Boero F (2010). The study of species in era of biodiversity: a tale of stupidity.. Diversity (open-access).

[178] Center for Marine Biodiversity.

[179] Zwanenburg KCT, Querbach K, Kenchington ELR, Frank K (2002).

[180] Radulovici AE, Archambault P, Dufresne F (2010). DNA barcoding for marine biodiversity: more than a trendy approach.. Diversity.

[181] Saunders GW (2008). A DNA barcode examination of the red algal family Dumontiaceae in Canadian waters reveals substantial cryptic species diversity. 1. The foliose *Dilsea-Neodilsea* complex and *Weeksia*.. Botany.

[182] Robba L, Russell SJ, Barker GL, Brodie J (2006). Assessing the use of the mitochondrial cox1 marker for use in DNA barcoding of red algae (Rhodophyta).. Am J Bot.

[183] Lindstrom S (2008). Cryptic diversity, biogeography and genetic variation in northeast Pacific species of Porphyra sensu lato (Bangiales, Rhodophyta).. J Appl Phycol.

[184] Worm B, Lotze HK, Jonsen I, Muir C, McIntyre AD (2010). The Future of Marine Animal Populations.. Life in the World's Oceans: Diversity, Distribution, and Abundance.

[185] Payne J, Andrews K, Chittenden C, Crossin G, Goetz F, McIntyre AD (2010). Tracking Fish Movements and Survival on the Northeast Pacific Shelf.. Life in the World's Oceans: Diversity, Distribution, and Abundance.

[186] Gradinger R, Bluhm BA, Hopcroft RR, Gebruk A, Kosobokova K, McIntyre AD (2010). Marine Life in the Arctic.. Life in the World's Oceans: Diversity, Distribution, and Abundance.

[187] Konar B, Iken K, Pohle G, Miloslavich P, Cruz-Motta JJ, McIntyre AD (2010). Surveying Nearshore Biodiversity.. Life in the World's Oceans: Diversity, Distribution, and Abundance.

[188] Vanden Berghe E, Stocks K, Grassle JF, McIntyre AD (2010). Data Integration: The Ocean Biogeographic Information System.. Life in the World's Oceans: Diversity, Distribution, and Abundance.

[189] Denman K, Forbes R, Mackas DL, Hill S, Sefton H (1985). Ocean ecology data report: British Columbia coastal waters, 29 June–10 July 1983.. Can Data Rep Hydrogr Ocean Sci.

[190] Forbes JR, Waters RE (1993). Phytoplankton species composition and abundance along the Pacific coast of Canada, 1979–1989. Volume 1.. Can Data Rep Hydrogr Ocean Sci.

[191] Forbes JR, Waters RE (1993). Phytoplankton species composition and abundance along the Pacific coast of Canada, 1979–1989. Volume 2.. Can Data Rep Hydrogr Ocean Sci.

[192] Hill S, Denman K, Mackas D, Sefton H (1982). Ocean ecology data report: coastal waters off southwest Vancouver Island. Spring and summer 1979.. Can Data Rep Hydrogr Ocean Sci.

[193] Hill S, Denman K, Mackas D, Sefton H (1982). Ocean ecology data report: coastal waters off southwest Vancouver Island. Spring and summer 1980.. Can Data Rep Hydrogr Ocean Sci.

[194] Hill S, Denman K, Mackas D, Sefton H, Forbes JR (1983). Ocean ecology data report: coastal waters off southwest Vancouver Island. Spring and summer 1981.. Can Data Rep Hydrogr Ocean Sci.

[195] Waters RE, Brown LN, Robinson MG (1992). Phytoplankton of Esquimalt Lagoon, British Columbia: comparison with west Vancouver Island and offshore waters.. Can Tech Rep Hydrogr Ocean Sci.

[196] Adams W (1975). Light intensity and primary productivity under sea ice containing oil. Beaufort Sea Technical Report 29.

[197] Bain H, Thomson D, Foy M, W. G (1977).

[198] Bursa AS (1969). *Kofoidinium arcticum*, a new dinoflagellate.. Phycologia.

[199] Bursa AS (1969). *Actiniscus canadensis* n. sp., *A. pentasterias* Ehrenberg v. *arcticus* n. var., *Pseudoactiniscus apentasterias* n. gen., n. sp., marine relicts in Canadian Arctic lakes.. J Protozool.

[200] Bursa AS (1969). *Dinamoebidium hyperboreum* spec. nov. in coastal plankton of Ellesmere Island, Northwest Territories, Canada.. Arct Alp Res.

[201] Bursa AS (1971). Biological oceanographic observations in Frobisher Bay. III. Phytoplankton tables, 1967.. Fish Res Board Can Tech Rep.

[202] Foy MG, Hsiao SIC (1976). Phytoplankton data from the Beaufort Sea, 1973 to 1975.. Fish Mar Serv Res Dev Tech Rep.

[203] Hsiao SIC, Beaufort Sea Project Tech Rep, editor (1976). Biological productivity of the southern Beaufort Sea: phytoplankton and seaweed studies..

[204] Hsiao SIC (1979). Phytoplankton and sea ice microalgal data from Frobisher Bay, 1971 to 1978.. Fish Mar Serv Data Rep.

[205] Hsiao SIC (1985). The growth of Arctic marine phytoplankton in Frobisher Bay.. Arctic.

[206] Hsiao SIC, Foy MG, Kittle DW (1977). Standing stock, community structure, species composition, distribution, and primary production of natural phytoplankton in the southern Beaufort Sea.. Can J Bot.

[207] Hsiao SIC, Pinkewycz N (1985). Arctic marine phytoplankton contributed to the sediments in Frobisher Bay.. Can Data Rep Fish Aquat Sci.

[208] Hsiao SIC, Trucco R (1980). Phytoplankton. In: A marine biological study of Brevoort Harbour and nearby waters of eastern Baffin Island.. Can Manuscr Rep Fish Aquat Sci.

[209] Lovejoy C, Legendre L, Martineau M-J, Bâcle J, von Quillfeldt CH (2002). Distribution of phytoplankton and other protists in the North Water.. Deep-Sea Res Pt II.

[210] MacLaren Atlantic Limited (1977).

[211] MacLaren Atlantic Limited (1978).

[212] MacLaren Marex Inc. (1979).

[213] Mann A, Acland FA (1925). Marine diatoms..

[214] Manton I, Sutherland J, Leadbeater BSC (1975). Four new species of choanoflagellates from Arctic Canada.. P R Soc B.

[215] Manton I, Sutherland J, Leadbeater BSC (1976). Further observations on the fine structure of marine collared flagellates (Choanoflagellata) from arctic Canada and west Greenland: species of *Parvicorbicula* and *Pleurasiga*.. Can J Bot.

[216] Manton I, Sutherland J, Oates K (1976). Arctic coccolithophorids: two species of *Turrisphaera* gen. nov. from West Greenland, Alaska, and the Northwest Passage.. P R Soc B.

[217] Poulin M (2009).

[218] Riedel A, Michel C, Poulin M, Lessard S (2003). Taxonomy and abundance of microalgae and protists at a first-year sea ice station near Resolute Bay, Nunavut, spring to early summer 2001.. Can Data Rep Hydrogr Ocean Sci.

[219] Różańska M, Poulin M, Gosselin M (2008). Protist entrapment in newly formed sea ice in the Coastal Arctic Ocean.. J Mar Syst.

[220] Seidenfaden G, Polunin N (1947). Marine phytoplankton.. Botany of the Canadian Eastern Arctic Part II Thallophyta and Bryophyta.

[221] Sekerak AD, Buchanan RA, Foy MG, Bain H, Walder GL (1979). Studies of plankton in northwest Baffin Bay and adjacent waters, July–October 1978..

[222] Sekerak AD, Thomson D, Bain H, Acreman J (1976).

[223] Thomson D, Woods S, Acreman J (1975). Marine ecology survey in the central portion of the Canadian Arctic islands 1974..

[224] Anderson JT, Roff JC, Gerrath J (1981). The diatoms and dinoflagellates of Hudson Bay.. Can J Bot.

[225] Bursa AS (1961). Phytoplankton of the *Calanus* Expeditions in Hudson Bay, 1953 and 1954.. J Fish Res Board Can.

[226] Bursa AS (1961). The annual oceanographic cycle at Igloolik in the Canadian Arctic. II. The phytoplankton.. J Fish Res Board Can.

[227] Daugbjerg N, Hansen LE, Skovgaard K, Østergarrd JB, Jørgensen M (1991). An investigation on some protist plankton groups from the marine waters around Igloolik Island, Northwest Territories, Canada.. Arctic biology course (1989, Ogloolik Island, Northwest Territories, Canada).

[228] Daugbjerg N, Vørs N, Søeborg B, Jensen D, Schurmann H, Steffensen JF, Curtis MA (1994). Preliminary results from a small scale survey of marine protists from northern Foxe Basin in the vicinity of Igloolik Island, June 1992.. Research on Arctic biology Igloolik, Northwest Territories, Canada, June 8th–July 8th 1992.

[229] Davidson VM (1931). Biological and oceanographic conditions in Hudson Bay. 5. The planktonic diatoms in Hudson Bay.. Contrib Can Biol Fish.

[230] Harvey M, Therriault JC, Simard N (1997). Late-summer distribution of phytoplankton in relation to water mass characteristics in Hudson Bay and Hudson Strait (Canada).. Can J Fish Aquat Sci.

[231] Percy JA, Grainger EH, Bunch JN, Hsiao SIC (1992). Oceanography and planktonic communities of two northern Quebec fjords.. Can Data Rep Fish Aquat Sci.

[232] Polunin N (1992). The flora of Akpatok Island, Hudson Strait.. J Bot.

[233] Simard N, Therriault J-C, Larouche P, Vézina A, Plourde J (1996). Données d'océanographie physique et biologique recueillies dans l'est et le nord de la baie d'Hudson et dans le détroit d'Hudson en août et septembre 1993.. Rapp Stat Can Sci Halieut Aquat.

[234] Vørs N (1993). Heterotrophic Amebas, Flagellates and Heliozoa from Arctic Marine Waters (North-West-Territories, Canada and West Greenland).. Polar Biol.

[235] Citarella G (1982). Contribution à l'étude des phytoplanctontes du détroit de Northumberland, Nouveau-Brunswick, Canada.. Hydrobiologia.

[236] Iselin C (1930). A report on the coastal waters of Labrador, based on explorations of the “Chance” during the summer of 1926.. Proc Am Acad Arts Sci.

[237] Lessard S (2009). Liste des espèces phytoplanctoniques présentes dans l'estuaire du Saint-Laurent.. Programme zonal de monitorage atlantique (PZMA) 1986–2008.

[238] Martin JL, LeGresley MM, Strain PM (2001). Phytoplankton monitoring in the Western Isles region of the Bay of Fundy during 1997–98.. Can Tech Rep Fish Aquat Sci.

[239] Martin JL, LeGresley MM, Strain PM (2006). Plankton monitoring in the Western Isles region of the Bay of Fundy during 1999–2000.. Can Tech Rep Fish Aquat Sci.

[240] Martin JL, LeGresley MM, Strain PM, Clement P (1999). Phytoplankton monitoring in the southwest Bay of Fundy during 1993–96.. Can Tech Rep Fish Aquat Sci.

[241] Oceanographic Laboratory of Edinburgh (1973). Continuous plankton records: a plankton atlas of the North Atlantic and the North Sea.. Bull Mar Ecol.

[242] St-Pierre J-F, Runge J, Joly P, de Lafontaine Y (1996). Données physiques, chimiques et biologiques sur le plancton du nord du golfe du Saint-Laurent. Partie I: juin 1989.. Rapp Stat Can Sci Halieut Aquat.

[243] Arseneau MJ, Archambault P, Goudreau P (2003). Effects de la pêche commerciale sur le gisement de pétoncles d'Islande (*Chlamys islandica*) de l'île Rouge dans l'estuaire du Saint-Laurent: évaluation des impacts sur le pétoncle et sur la communauté benthique associée.. Rapp Tech Can Sci Halieut Aquat.

[244] Atkinson EG, Wacasey JW (1989). Benthic invertebrates collected from Hudson Bay, Canada, 1953 to1965.. Can Data Rep Fish Aquat Sci.

[245] Atkinson EG, Wacasey JW (1989). Benthic invertebrates collected from the western Canadian Arctic, 1951 to 1985.. Can Data Rep Fish Aquat Sci.

[246] Barrie JD (1979). Diversity of marine benthic communities from nearshore environments on the Labrador and Newfoundland coasts.

[247] Bourget E, Messier D (1983). Macrobenthic density, biomass, and fauna of intertidal and subtidal sand in a Magdalen Islands lagoon, Gulf of St. Lawrence.. Can J Zool.

[248] Bourque M (2008).

[249] Brinkhurst RO (1987). Distribution and abundance of macroscopic benthic infauna from the continental shelf off southwestern Vancouver Island, British Columbia, Canada.. Can Tech Rep Hydrogr Ocean Sci.

[250] Burd BJ (1990). Vancouver Harbour and Burrard Inlet benthic infaunal sampling program, October 1987.. Can Tech Rep Hydrogr Ocean Sci.

[251] Burd BJ, Brinkhurst RO (1987). Macrobenthic infauna from Hecate Strait, British Columbia.. Can Tech Rep Hydrogr Ocean Sci.

[252] Burd BJ, Moore D, Brinkhurst RO (1987). Distribution and abundance of macrobenthic infauna from Boundary and Mud Bays near the British Columbia/U.S. border.. Can Tech Rep Hydrogr Ocean Sci.

[253] Caddy JF, Amaratunga T, Dadswell MJ, Edelstein T, Linkletter LE (1977). 1975 Northumberland Strait Project, Part I: Benthic fauna, flora, demersal fish, and Sedimentary Data.. Fish Mar Serv Manus Rep.

[254] Cross SF, Brinkhurst RO (1991). Spatial distribution of macrobenthic infauna in Burrard Inlet: November 1989.. Can Data Rep Hydrogr Ocean Sci.

[255] Desrosiers G, Savenkoff C, Olivier M, Stora G, Juniper K (2000). Trophic structure of macrobenthos in the Gulf of St. Lawrence and on the Scotian Shelf.. Deep-Sea Res Pt II.

[256] Hughes RN, Peer DL, Mann KH (1972). Use of multivariate analysis to identify functional components of the benthos in St. Margaret's Bay, Nova Scotia.. Limnol Oceanogr.

[257] Knox D (1980). Identification and enumeration of benthic organisms in samples from Canso Strait and Chedabucto Bay..

[258] O'Connell B (1978).

[259] Peer DL, Wildish AJ, Hines J, Dadswell MJ (1980). Sublittoral macro-infauna of the Lower Bay of Fundy.. Can Tech Rep Fish Aquat Sci.

[260] Prena J, Rowell TW, Schwinghamer P, Gilkinson K, Gordon DCJ (1996). Grand Banks otter trawling impact experiment: I. Site selection process with a description of macrofaunal communities.. Can Tech Rep Fish Aquat Sci 2094 Can Tech Rep Fish Aquat Sci 2094..

[261] Ramey PA, Snelgrove PVR (2003). Spatial patterns in sedimentary macrofaunal communities on the south coast of Newfoundland in relation to surface oceanography and sediment characteristics.. Mar Ecol Prog Ser.

[262] Stewart PL, Kendrick PA, Levy HA, Robinson TL, Lee K (2002). Soft bottom benthic communities in Sydney Harbour, Nova Scotia. 2. 2000 survey. Distribution and relation to sediments and contamination.. Can Tech Rep Fish Aquat Sci.

[263] Wacasey JW, Atkinson EG, Derick L, Weinstein A (1977). Zoobenthos data from the southern Beaufort Sea, 1971–1975.. Fish Mar Serv Data Rep.

[264] Wacasey JW, Atkinson EG, Glasspoole L (1979). Zoobenthos data from upper Frobisher Bay, 1967–1973.. Can Data Rep Fish Aquat Sci.

[265] Wacasey JW, Atkinson EG, Glasspoole L (1980). Zoobenthos data from inshore stations of upper Frobisher Bay, 1969–1976.. Can Data Rep Fish Aqua Sci.

[266] Wacasey JW, Atkinson EG, Kinlough L (1976). Zoobenthos data from James Bay, 1959, 1974.. Fish Mar Serv Res Dev Tech Rep.

[267] Wildish DJ (1977). Sublittoral macro-infauna of Musquash estuary.. Fish Mar Serv Manus Rep.

[268] Wildish DJ, Wildish AJ, Akagi HM (1977). Sub-littoral macro-infauna of St. Croix estuary.. Fish Mar Serv Manus Rep.

[269] Jean Y, Peden AE, McAllister DE (1981). English, French and Scientific Names of Pacific Fish of Canada.. British Columbia Provincial Museum Heritage Record.

[270] Froese R, Pauly D (2009). FishBase. World Wide Web electronic publication.. http://www.fishbase.org.

