# Supplementary material for: From Sea to Sea: Canada's Three Oceans of Biodiversity
Source: PLoS One. 2010 Aug 31;5(8):e12182. doi: 10.1371/journal.pone.0012182 (PMC2930843; doi:10.1371/journal.pone.0012182)
Supplement: Text S1 — Territorial sea data is from L. Pruett and J. Cimino, unpublished data, Global Maritime Boundaries Database (GMBD), Veridian - MRJ Technology Solutions, (Fairfax, Virginia, January, 2000) (excluding Caspian sea and 2,867,050 km2 of disputed territorial). Territorial Sea is defined under the United Nations Convention on the Law of the Sea (UNCLOS) as the 12-nautical mile zone from the baseline or low-water line along the coast. The coastal State's sovereignty extends to the territorial sea, including its sea-bed, subsoil, and air space above it. Foreign vessels are allowed “innocent passage” through those waters. Even though the established limit for a territorial sea is 12 nautical miles, some countries claim larger areas. Territorial seas with overlapping claims from different countries are shown separately as disputed territorial seas. UNCLOS is an international agreement that sets conditions and limits on the use and exploitation of the oceans. This Convention also sets the rules for the maritime jurisdictional boundaries of the different member states. The UNCLOS was opened for signature on 10 December 1982 in Montego Bay, Jamaica, and it entered into force on 16 November 1994. As of January 2000, there are 132 countries that have ratified UNCLOS. Given the uncertainties surrounding much of the delimitation of the territorial seas, these figures should be used with caution. Please refer to the original source for further information on the variables and collection methodologies or to the following Web site: http://earthtrends.wri.org/. For more information in UNCLOS please refer to the United Nations Web page at: http://www.un.org/Depts/los/index.htm. (0.03 MB DOC) [file pone.0012182.s001.doc]

**Text S1**: Territorial sea data is from L. Pruett and J. Cimino, unpublished data, Global Maritime Boundaries Database (GMBD), Veridian - MRJ Technology Solutions, (Fairfax, Virginia, January, 2000) (excluding Caspian sea and 2,867,050 km^2^ of disputed territorial). Territorial Sea is defined under the United Nations Convention on the Law of the Sea (UNCLOS) as the 12-nautical mile zone from the baseline or low-water line along the coast. The coastal State's sovereignty extends to the territorial sea, including its sea-bed, subsoil, and air space above it. Foreign vessels are allowed "innocent passage" through those waters. Even though the established limit for a territorial sea is 12 nautical miles, some countries claim larger areas. Territorial seas with overlapping claims from different countries are shown separately as disputed territorial seas. UNCLOS is an international agreement that sets conditions and limits on the use and exploitation of the oceans. This Convention also sets the rules for the maritime jurisdictional boundaries of the different member states. The UNCLOS was opened for signature on 10 December 1982 in Montego Bay, Jamaica, and it entered into force on 16 November 1994. As of January 2000, there are 132 countries that have ratified UNCLOS. Given the uncertainties surrounding much of the delimitation of the territorial seas, these figures should be used with caution. Please refer to the original source for further information on the variables and collection methodologies or to the following Web site: http://earthtrends.wri.org/. For more information in UNCLOS please refer to the United Nations Web page at: <http://www.un.org/Depts/los/index.htm>.
