# Supplementary material for: From Sea to Sea: Canada's Three Oceans of Biodiversity
Source: PLoS One. 2010 Aug 31;5(8):e12182. doi: 10.1371/journal.pone.0012182 (PMC2930843; doi:10.1371/journal.pone.0012182)
Supplement: Text S2 — Coastal length data are based on the World Vector Shoreline, United States Defense Mapping Agency, 1989. Figures were calculated by L. Pruett and J. Cimino, unpublished data, Global Maritime Boundaries Database (GMBD), Veridian - MRJ Technology Solutions, (Fairfax, Virginia, January, 2000). (0.02 MB DOC) [file pone.0012182.s002.doc]

**Text S2**: Coastal length data are based on the World Vector Shoreline, United States Defense Mapping Agency, 1989. Figures were calculated by L. Pruett and J. Cimino, unpublished data, Global Maritime Boundaries Database (GMBD), Veridian - MRJ Technology Solutions, (Fairfax, Virginia, January, 2000).
