# Supplementary material for: From Sea to Sea: Canada's Three Oceans of Biodiversity
Source: PLoS One. 2010 Aug 31;5(8):e12182. doi: 10.1371/journal.pone.0012182 (PMC2930843; doi:10.1371/journal.pone.0012182)
Supplement: Table S1 — Numbers of marine zooplankton by family and species. (0.11 MB DOC) [file pone.0012182.s003.doc]

**Table S1. Numbers of marine zooplankton by family and species.** This list was compiled for the three Canadian ocean provinces and two other regions, the Hudson Bay System and the St. Lawrence Marine System.

|  |  |  | **Western Canada** | | **Canadian Arctic** | | **Hudson**  **Bay** | | **Eastern Canada** | | **St. Lawrence**  **Marine** | |
| --- | --- | --- | --- | --- | --- | --- | --- | --- | --- | --- | --- | --- |
| **Phylum** | **Class** | **Order** | Families | Species | Families | Species | Families | Species | Families | Species | Families | Species |
| Cnidaria | Hydrozoa | Anthoathecatae | 10 | 18 | 8 | 18 | 6 | 12 | 6 | 19 | 5 | 9 |
|  |  | Leptothecatae | 5 | 11 | 4 | 5 | 3 | 4 | 7 | 15 | 5 | 5 |
|  |  | Siphonophorae | 6 | 18 | 2 | 3 | 1 | 1 | 4 | 7 | 3 | 3 |
|  |  | Trachymedusae | 3 | 6 | 2 | 7 | 2 | 2 | 4 | 7 | 1 | 1 |
|  |  | Narcomedusae | 3 | 4 | 1 | 3 | 1 | 1 | 1 | 2 | 2 | 2 |
|  | Scyphozoa | Stauromedusae | 0 | 0 | 0 | 0 | 1 | 1 | 2 | 5 | 2 | 5 |
|  |  | Coronatae | 2 | 3 | 1 | 1 |  |  |  |  | 1 | 1 |
|  |  | Semaeostomeae | 3 | 3 | 1 | 1 | 2 | 2 | 3 | 5 | 2 | 4 |
| Ctenophora | Tentaculata | Cydippida | 2 | 4 | 2 | 3 | 1 | 1 | 2 | 2 | 2 | 2 |
|  |  | Lobata | 1 | 1 | 1 | 1 |  |  | 1 | 1 | 1 | 1 |
|  | Nuda | Berioda | 1 | 2 | 1 | 1 | 1 | 1 | 1 | 1 | 1 | 2 |
| Mollusca | Gastrodopoda | Thecosomata | 5 | 7 | 1 | 1 | 1 | 2 | 1 | 2 | 1 | 2 |
|  |  | Gymnosomata | 4 | 5 | 1 | 1 | 1 | 1 | 2 | 3 | 1 | 1 |
|  |  | Neotaenioglossa | 2 | 3 |  |  |  |  |  |  |  |  |
| Annelida | Polychaeta | Aciculata | 5 | 16 | 11 | 17 | 2 | 4 | 7 | 27 | 19 | 60 |
|  |  | Canalipalpata | 1 | 1 | 9 | 11 | 18 | 24 | 7 | 14 | 11 | 36 |
| Arthropoda | Branchiopoda | Diplostraca | 1 | 1 | 2 | 4 | 2 | 5 | 1 | 6 | 2 | 6 |
|  | Ostracoda | Halocyprida | 1 | 45 | 2 | 7 |  |  | 3 | 6 | 2 | 3 |
|  |  | Myodocopida | 2 | 1 | 1 | 1 |  |  |  |  |  |  |
|  |  | Podocopida | 1 | 1 | 1 | 10 |  |  | 1 | 1 |  |  |
|  | Maxillopoda | Calanoida | 24 | 185 | 24 | 104 | 14 | 35 | 22 | 96 | 16 | 48 |
|  |  | Harpacticoida | 3 | 4 | 15 | 65 | 10 | 25 |  |  | 14 | 25 |
|  |  | Cyclopoida | 1 | 4 | 3 | 7 | 2 | 5 | 2 | 5 | 2 | 7 |
|  |  | Poecilostomatoida | 4 | 11 | 3 | 6 | 1 | 1 | 17 | 52 | 2 | 5 |
|  | Maxillopoda | Monstrilloida | 1 | 3 | 1 | 1 | 1 | 1 | 1 | 5 | 1 | 6 |
|  |  | Siphonostomatoida | 1 | 1 | 0 | 0 |  |  | 1 | 1 | 4 | 5 |
|  |  | Mormonilloida | 1 | 2 | 1 | 2 |  |  |  |  |  |  |
|  |  | Pedunculata | 0 | 0 |  |  | 1 | 1 | 1 | 4 | 2 | 4 |
|  | Malacostraca | Mysida | 1 | 17 | 1 | 11 | 1 | 10 | 1 | 9 | 2 | 23 |
|  |  | Isopoda | 1 | 1 | 2 | 3 | 1 | 1 | 5 | 6 | 5 | 6 |
|  |  | Amphipoda | 17 | 53 | 21 | 61 | 14 | 34 | 18 | 41 | 20 | 64 |
|  |  | Euphausiacea | 2 | 18 | 1 | 5 | 1 | 4 | 1 | 7 | 1 | 5 |
|  |  | Decapoda | 3 | 8 | 4 | 5 | 3 | 4 | 9 | 16 | 11 | 38 |
|  |  | Lophogastrida | 2 | 4 |  |  |  |  |  |  |  |  |
| Chaetognatha | Sagittoidea | Phragmophora | 1 | 2 | 1 | 2 | 1 | 1 | 1 | 1 | 1 | 2 |
|  |  | Aphragmophora | 2 | 6 | 1 | 2 | 1 | 3 | 1 | 7 | 1 | 3 |
| Chordata | Appendicularia | Copelata | 2 | 5 | 2 | 3 | 2 | 4 | 2 | 4 | 2 | 4 |
|  | Thaliacea | Salpida | 1 | 5 | 0 | 0 |  |  | 1 | 4 | 1 | 1 |
|  |  | Doliolida | 2 | 2 |  |  |  |  |  |  |  |  |
|  |  | TOTAL ZOOPLANKTON | 127 | 481 | 131 | 372 | 95 | 190 | 136 | 381 | 146 | 389 |

Much of the information for this section is based on a thorough synopsis of zooplankton from the three Canadian ocean provinces (Eastern Canada, Western Canada, and the Canadian Arctic) provided by [89] based on a synthesis of materials collected before 1970 to depths less than 200 m, with additions from:[S1,S2] for the Atlantic; [41] in the St. Lawrence Marine System (SLMS);[232,233,S3,S4,S5], and DFO MERICA monitoring program (2003**–**2006) for the Hudson Bay, Hudson Strait, and Fox Basin area; a comprehensive list of the holoplanktonic zooplankton species that DFO researchers have found in the Western Canada and Canadian Arctic regions during the past 30 years; and [S6,S7,S8], and data from [S6,S7,S8,S9] for the western Arctic.

**REFERENCES.**

S1. Frost BW (1989) A taxonomy of the marine calanoid copepod genus *Pseudocalanus*. Can J Zool 67: 551.

S2. Tremblay MJ, Anderson J (1984) Annotated species list of marine planktonic copepods occurring on the shelf and upper slope of the Northwest Atlantic. Can Spec Publ Fish Aquat Sci 69: 1-12.

S3. Grainger EH, McSwenn S (1976) Marine zooplankton and some physical-chemical features of James Bay related to La Grande hydro-electric development. Fish Mar Serv Res Dev Tech Rep 650: 94 p.

S4. Hudon C, Crawford RE, Ingram RG (1993) Influence of physical forcing on the spatial-distribution of marine fauna near Resolution Island (Eastern Hudson Strait). Mar Ecol Prog Ser 92: 1-14.

S5. Rochet M, Grainger EH (1988) Community structure of zooplankton in eastern Hudson Bay. Can J Zool 66: 1626-1630.

S6. Grainger EH, Grohe C (1975) Zooplankton data from the Beaufort Sea 1951 to 1975. Fish Res Board Can Tech Rep 591: 54 p.

S7. Kosobokova KN, Hopcroft RR (2010) Diversity and vertical distribution of mesozooplankton in the Arctic’s Canada Basin. Deep-Sea Res Pt II 57: 96-110.

S8. Mohammed AA, Grainger EH (1974) Zooplankton data from the Canadian Arctic Archipelago,1962. Can Tech Rep Fish Aquat Sci 460: 135 p.

S9. Hopky GE, Lawrence MJ, Chiperzak DB (2004) NOGAP B2: Zooplankton Data from the Canadian Beaufort Sea Shelf, 1986. Can Data Rep Fish Aquat Sci 923: 112 p.
