# Supplementary material for: From Sea to Sea: Canada's Three Oceans of Biodiversity
Source: PLoS One. 2010 Aug 31;5(8):e12182. doi: 10.1371/journal.pone.0012182 (PMC2930843; doi:10.1371/journal.pone.0012182)
Supplement: Table S2 — Occurrence, status, and demographic trends of species and populations of marine mammals (Eastern Canada, Canadian Arctic, and Western Canada provinces). (0.10 MB DOC) [file pone.0012182.s004.doc]

**Table S2. Occurrence, status, and demographic trends of species and populations of marine mammals** (Eastern Canada, Canadian Arctic, and Western Canada provinces). Population status can be EX=extirpated, EN=endangered, TH=threatened, NT=near threatened, LC=least concern, SC=special concern, V=vulnerable, NR=not at risk, DD=data deficient, or NA=Not available. Population trends can be DEC=decreasing, INC=increasing, STA=stable, or ?=unknown. Population (COSEWIC) and species (IUCN) status and trends were obtained from [www.cosewic.gc.ca](http://www.cosewic.gc.ca/) and [www.iucnredlist.org](http://www.iucnredlist.org/), respectively. Boldface for status and trend indicates that the species was historically (status) or currently (trend) harvested.

| **Species** | **IUCN** | | **Occurrence and population status** | | | **Source** |
| --- | --- | --- | --- | --- | --- | --- |
|  | Status | Trend | Eastern Canada | Canadian Arctic | Western Canada |  |
| Atlantic walrus, *Odobenus rosmarus rosmarus* | **DD** | **?** | EX | SC | SC-(NAI) | [S10,S11,S12] |
| Atlantic white-sided dolphin, *Lagenorhynchus acutus* | **LC** | **?** | NR |  |  | [S11,S12,S13] |
| Baird's beaked whale, *Berardius bairdii* | DD | ? |  |  | NR | [S12,S13] |
| Bearded seal, *Erignatus barbatus* | **LC** | **STA** | DD | DD | DD(NAI) | [S11,S12,S44] |
| Beluga, *Delphinapterus leucas* | **NT** | **?** | TH | 2EN, 1TH, 2SC, 1NR | 1EN^1^ | [S12,S13,S15,S16] |
| Blainville's beaked whale, *Mesoplodon densirostris* | DD | ? | NR |  |  | [S11,S12,S13] |
| Bottlenose dolphin, *Tursiops truncatus* | LC | ? | NR |  |  | [S11,S12] |
| Blue whale, *Balaenoptera musculus* | **EN** | INC | EN | EN | EN | [S11,S12,S13] |
| Bowhead whale, *Balaena mysticetus* | **LC** | **INC** |  | 2TH, 1SC | SC (NAI) | [S17] |
| California sea lion, *Zalophus californianus* | **LC** | INC |  |  | NR | [S12] |
| Common minke whale, *Balaenoptera acutorostrata* | **LC** | **STA** | NR | NR | NR | [S11,S12,S13] |
| Cuvier's beaked whale, *Ziphius cavirotris* | LC | ? | NR |  | NR | [S12] |
| Dall's porpoise, *Phocoenoides dalli* | **LC** | ? |  |  | NR | [S12] |
| Dwarf sperm whale, *Kogia sima* | DD | ? |  |  | DD | [S11,S12] |
| False killer whale, *Pseudorca crassidens* | DD | ? |  |  | NR | [S11,S12] |
| Fin whale, *Balaenoptera physalus* | **EN** | ? | SC | TH | TH | [S11,S12,S13] |
| Gray whale, *Eschrichtius robustus* | **LC** | **STA** | EX | EX (Atl), SC (Pac) | SC | [S11,S12,S13] |
| Grey seal, *Halichoerus grypus* | **LC** | **INC** | NR |  |  | [S12] |
| Harbour porpoise, *Phocoena phocoena* | **LC** | ? | SC | SC | SC | [S11,S12,S13] |
| Freshwater harbour seal, *Phoca vitulina mellonae* | **LC** | **STA** |  | EN |  | [S11,S12] |
| Harbour seal, *Phoca vitulina* | **LC** | **STA** | NR | NR | NR | [S11,S12] |
| Harp seal, *Phoca groenlandica* | **LC** | **INC** | NA^2^ | NA^2^ |  | [S11,S12,S18] |
| Hooded seal, *Cystophora cristata* | **VU** | **DEC** | NR | NR |  | [S11,S12] |
| Hubb's beaked whale, *Mesoplodon carlhubbis* | DD | ? |  |  | NR | [S12] |
| Killer whale, *Orcinus orca* | DD | ? | 1SC | 1TH(Pac); 1SC(Atl) | 1EN, 3TH, | [S11,S12,S13] |
| Humpback whale, *Megaptera novaeangliae* | **LC** | INC | NR | TH | TH | [S11,S12,S13, S19] |
| Spotted seal, *Phoca largha* | DD | ? |  | NA^3^ | NA^3^ (NAI) | [S12] |
| Narwhal, *Monodon monoceros* | **NT** | **?** |  | SC |  | [S11,S12,S13] |
| Northern bottlenose whale, *Hyperodon ampullatus* | **DD** | ? | 1EN, 1NR | NR |  | [S11,S12,S13] |
| Northern elephant seal, *Mirounga angustirostris* | **LC** | INC |  |  | NR | [S12] |
| Northern fur seals, *Callorhiynus ursinus* | **VU** | **DEC** |  | TH (NAI) | TH | [S12] |
| Northern right-whale dolphin, *Lissodelphis borealis* | LC | ? |  |  | NR | [S12] |
| White-sided dolphin, *Lagenorhynchus obliquidens* | LC | ? |  |  | NR | [S12] |
| Polar bear, *Ursus maritimus* | **VU** | **DEC** | SC | SC |  | [S11,S12] |
| Pygmy sperm whale, *Kogia breviceps* | DD | ? | NR |  | NR | [S11,S12,S13,S20,S21] |
| Ribbon seal, *Histriophoca fasciata* | DD | ? |  | ?^3^ | ?(NAI) | [S12] |
| North Atlantic right whale, *Eubalaena glacialis* | **EN** | ? | EN |  |  | [S11,S12,S13] |
| North Pacific right whale, *Eubalaena japonica* | **EN** | ? |  |  | EN | [S11,S12,S13] |
| Ringed seal, *Pusa hispida* | **LC** | **?** | NR | NR | NR(NAI) | [S12] |
| Risso's dolphin, *Grampus griseus* | LC | ? | NR |  | NR | [S11,S12,S13,S19] |
| Sea otter, *Enhydra lutris* | **EN** | STA |  |  | SC | [S12] |
| Sei whale, *Balaenoptera borealis* | **EN** | ? | DD | EN | EN | [S11,S12,S13] |
| Short-beaked common dolphin, *Delphinus delphis* | LC | ? | NR |  | NR | [S11,S12,S13] |
| Short-finned pilot whale, *Globicephala macrorhynchus* | **DD** | ? |  |  | NR | [S12] |
| Sowerby's beaked whale, *Mesoplodon bidens* | DD | ? | SC |  |  | [S11,S12,S13] |
| Sperm whale, *Physeter macrocephalus* | **VU** | ? | NR | NR | NR | [S11,S12,S13]] |
| Stejneger's beaked whale, *Mesoplodon stejneri* | DD | ? |  |  | NR | [S12] |
| Steller sea Lion, *Eumetopias jubatus* | **EN** | **DEC** |  |  | SC | [S12] |
| Striped dolphin, *Stenella coeruleoalba* | LC | ? | NR |  | NR | [S11,S12,S13,S20] |
| True's beaked whale, *Mesoplodon mirus* | DD | ? | NR |  |  | [S11,S12,S13] |
| Long-finned pilot whale, *Globicephala melas* | **DD** | ? | NR |  |  | [S12] |
| White-beaked dolphin, *Lagenorhynchus albirostris* | LC | ? | NR |  |  | [S11,S12,S13] |
| Total of species |  |  | 30 | 24 | 37 |  |

^1^Cook Inlet population, southern Alaska (Endangered as per US Marine Mammal Protection Act)

^2^This population was not examined by COSEWIC, but is stable at ~ 6 million individuals

^3^Species not examined by COSEWIC; current population size and trends unknown

^4^Subspecies

**REFERENCES.**

S10. COSEWIC (2006) COSEWIC assessment and update status report on Atlantic walrus *Odobenus rosmarus rosmarus* in Canada. Ottawa: Committee on the Status of Endangered Wildlife in Canada: 85 p.

S11. Prescott J, Richard P (1996) Mammifères du Québec et de l'est du Canada. Waterloo, Ontario: Editions Michel Quintin.

S12. Reeves RR, Stewart BS, Clapham PJ, Powell JA (2002) Marine mammals of the world. New York: Chanticleer Press, Inc.

S13. Carwardine M (2002) Whales, dolphins and porpoises. New York: Dorling Kindersley Publishing. 288 p.

S14. COSEWIC (2008) Update COSEWIC status report on bearded seal *Erignatus barbatus* in Canada. Ottawa: Committee on the Status of Endangered Wildlife in Canada: 36 p.

S15. COSEWIC (2004) COSEWIC assessment and update status report on the beluga whale *Delphinapterus leucas* in Canada. Ottawa: Committee on the Status of Endangered Wildlife in Canada,: 70 p.

S16. Hobbs R, Shelden KEW, Rugh DJ, SA N (2008) 2008 Status review and extinction risk assessment of Cook Inlet belugas (*Delphinapterus leucas*) Alaska Fisheries Science Center, NOAA, National Marine Fisheries Service. 116 p.

S17. COSEWIC (2009) COSEWIC Updated status report on the bowhead whale, *Balaena mysticetus*, Eastern Canada - West Greenland population, Bering-Chukchi-Beaufort population, in Canada. Ottawa: Committee on the Status of Endangered Wildlife in Canada: 46 p.

S18. DFO, Canadian Department of Fisheries and Oceans (2005) Stock Assessment of Northwest Atlantic Harp Seals (*Pagophilus groenlandicus*). DFO Can Sci Advis Sec Sci Advis Rep 2005/037: 1-12.

S19. Palka DL (2006) Summer abundance estimates of cetaceans in US North Atlantic Navy Operating Areas. Northeast Fish Sci Cent Ref Doc 06-03: 1-41.

S20. Lesage V, Gosselin J-F, Hammill MO, Kingsley MCS, Lawson JW (2007) Ecologically and Biologically Significant Areas (EBSAs) in the Estuary and Gulf of St. Lawrence - A marine mammal perspective. Can Sci Adv Sec Res Doc 2007/046: 1-94.

S21. Measures L, Roberge B, Sears R (2004) Stranding of a pygmy sperm whale (*Kogia breviceps*) in the Northern Gulf of St. Lawrence, Canada. Can Field Nat 118: 495-498.
