# Supplementary material for: From Sea to Sea: Canada's Three Oceans of Biodiversity
Source: PLoS One. 2010 Aug 31;5(8):e12182. doi: 10.1371/journal.pone.0012182 (PMC2930843; doi:10.1371/journal.pone.0012182)
Supplement: Alternative Language Abstract S1 — Abstract in French - Résumé en français. French translation of the abstract by Philippe Archambault. (0.02 MB DOC) [file pone.0012182.s005.doc]

## Résumé

La compréhension et l’évaluation de la biodiversité au sein des écosystèmes marins représentent un défi de taille, mais se voient également comme une nécessité. L’objectif du présent manuscrit vise à compiler et résumer la biodiversité marine présente dans les trois océans canadiens. Bien entendu, cette compilation n’est pas définitive et risque de potentiellement changer dans les années futures. Le Canada possède la plus grande côte du littoral au monde et se compose d’un large éventail de provinces biogéographiques et d’écorégions (ex : tempérée à couvert de glace important). Cet aspect peu négligeable caractérise ainsi le Canada comme une « zone d’étude » intéressante pour l’étude de la biodiversité marine. Les groupes taxinomiques représentés dans cette synthèse incluent ainsi les microbes, le phytoplancton, les macroalgues, le zooplancton, la faune endobenthique, les poissons et les mammifères marins. Le nombre minimal d’espèces/taxon compilé pour les trois océans canadiens dans cette synthèese est de 15 988. Toutefois, cette synthèse n’est pas exhaustive et le nombre de taxa reporté dans les océans canadiens est significativement sous-estimé. Cela s’explique en partie, tout d’abord par le manque d’informations publiées dans la littérature. Deuxièmement, la diversité de certains habitats n’a pu être compilée pour certains taxa tels que l’habitat intertidal rocheux et les zones profondes. De plus, la compilation des données est basée sur différent type d’étude (études à court terme, des programmes de recherches spécifiques ou activités de monitorage à long terme) limitant ainsi la résolution spatiale. Troisièmement, la biodiversité des organismes de grande taille est beaucoup mieux connue que celle des plus petites espèces. Finalement, la contrainte la plus importante de ce manuscrit demeure la disponibilité des données, puisque la compilation est dictée par l’enthousiasme plus ou moins important des scientifiques face à la biodiversité. Mentionnons d'ailleurs que le nombre de taxonomistes et de spécialistes dans le domaine marin a diminué à un taux alarmant, principalement dû au manque de formation et des opportunités de carrière disponible dans ce domaine. En prenant en compte l’ensemble de ces difficultés encourues lors de la compilation des données des espèces/taxa retrouvées dans les trois océans canadiens, nous espérons que cette revue, de pair avec le Réseau canadien sur la santé des océans (CHONe), guidera la façon de mieux résumer ce genre d’information. Un effort majeur doit être entrepris au Canada afin d’établir un niveau de référence adéquate de la biodiversité marine canadienne de tous les groupes taxinomique, surtout dans une vision de compréhension et de préservation de cette partie de notre héritage naturel.
